# Digital Gene Expression Tag Profiling Analysis of the Gene Expression Patterns Regulating the Early Stage of Mouse Spermatogenesis

**DOI:** 10.1371/journal.pone.0058680

**Published:** 2013-03-15

**Authors:** Xiujun Zhang, Lili Hao, Lijun Meng, Meiling Liu, Lina Zhao, Fen Hu, Cunbao Ding, Yang Wang, Baoling He, Yuxin Pan, Wei Fang, Jing Chen, Songnian Hu, Mengchun Jia

**Affiliations:** 1 College of Life Sciences, Hebei United University, Tangshan, Hebei, China; 2 Department of Reproductive Endocrinology, National Research Institute for Family Planning, Beijing, China; 3 CAS Key Laboratory of Genome Sciences and Information, Beijing Institute of Genomics, Chinese Academy of Sciences, Beijing, China; 4 Department of Environment and Chemical Engineering, Tangshan College, Tangshan, Hebei, China; Institute of Zoology, Chinese Academy of Sciences, China

## Abstract

Detailed characterization of the gene expression patterns in spermatogonia and primary spermatocytes is critical to understand the processes which occur prior to meiosis during normal spermatogenesis. The genome-wide expression profiles of mouse type B spermatogonia and primary spermatocytes were investigated using the Solexa/Illumina digital gene expression (DGE) system, a tag based high-throughput transcriptome sequencing method, and the developmental processes which occur during early spermatogenesis were systematically analyzed. Gene expression patterns vary significantly between mouse type B spermatogonia and primary spermatocytes. The functional analysis revealed that genes related to junction assembly, regulation of the actin cytoskeleton and pluripotency were most significantly differently expressed. Pathway analysis indicated that the Wnt non-canonical signaling pathway played a central role and interacted with the actin filament organization pathway during the development of spermatogonia. This study provides a foundation for further analysis of the gene expression patterns and signaling pathways which regulate the molecular mechanisms of early spermatogenesis.

## Introduction

Spermatogenesis, the process by which germ stem cells (Type A spermatogonia) develop into mature spermatozoa, includes three phases: spermatocytogenesis (mitosis), meiosis and spermiogenesis. Germ stem cells divide mitotically to replace themselves and produce the cells which subsequently differentiate (Type B spermatogonia). After a further mitotic division, type B spermatogonia divide mitotically into primary spermatocytes, followed by a meiotic division to generate secondary spermatocytes which eventually generate early and late spermatids [1]. Unraveling the molecular mechanisms which regulate mitotic and meiotic cell division in mammalian germ cells may help to understand the genetic basis of spermatogenesis. Much of the research conducted on the transcriptional regulation of spermatogenesis in the last two decades has focused on individual transcription factors, and most of these studies have relied solely on the altered phenotypes of knockout mice to assess the function of transcription factors [2]. However, the physiological links between different transcription factors at the various stages of the seminiferous epithelial cycle are largely unknown [3]. Furthermore, the mechanisms by which these genes and their proteins regulate different facets of spermatogenesis, such as the germ cell cycle, spermatogonial proliferation and renewal, germ cell apoptosis, meiosis, cell adhesion, junction restructuring, germ cell migration and other biochemical and morphological events pertinent to spermiogenesis remain unexplored [4].

A variety of different genes are involved in the processes which regulate spermatogenesis, and over 30 markers for different stages of germ cells have been identified in the rodent testis [5]. Rossi *et al.* examined gene expression during the mitotic and meiotic stages of male germ cell differentiation, and grouped the differentially expressed genes (DEGs) into functional clusters [6]. Additionally, other studies have characterized testis cell-specific transcripts and their primary functions in different types of testis cells, such as Leydig cells, Sertoli cells, myoid cells, premeiotic germ cells, meiotic and postmeiotic germ cells [7]. Although these general gene expression patterns are meaningful when the testis is regarded as an entire tissue, detailed characterization of the gene expression patterns which occur at each stage of germ cell differentiation are necessary in order to fully understand the molecular mechanisms of spermatogenesis.

To investigate the genetic mechanisms regulating early spermatogenesis, the global gene expression profiles of type B spermatogonia and primary spermatocytes prior to meiosis were identified using the Solexa/Illumina DGE system, a tag-based massively parallel transcriptome sequencing method on the Illumina platform [8].

## Materials and Methods

### Cell culture

GC-1spg and GC-2spd (ts) cells (ATCC, Manassas, VA, USA) were maintained in DMEM medium containing 10% FBS and 1.5 g/L sodium bicarbonate. GC-1spg cells were created by transformation of 10 day-old mouse type B spermatogonia with pSV3-neo, and have the characteristics of the stage between type B spermatogonia and primary spermatocytes. GC-2spd (ts) cells were created by transformation of 6 week-old mouse spermatocytes with SV40 large T antigen. The cells have lost their differentiation potential, and are currently arrested at a premeiotic stage.

### RNA extraction, library construction and sequencing

Total RNA was extracted from GC-1spg and GC-2spd (ts) cells using TRIzol® (Invitrogen, Carlsbad, CA, USA) and incubated with 10 U DNaseI (Takara, Dalian, China) for 30 min at 37°C to remove genomic DNA. RNA quality and quantity were determined by measuring the 260/280 nm absorbance ratio using a Nanodrop® ND-1000 spectrophotometer (LabTech, Holliston, MA, USA). The samples had an average RNA Integrity Number (RIN) value of 8.9 according to Labon-chip analysis (2100 Bioanalyzer; Agilent Technologies, Santa Clara, CA, USA).

The main reagents and instruments used for RNA library construction and deep sequencing were the Illumina Gene Expression Sample Prep Kit, Illumina Sequencing Chip (flowcell), Illumina Cluster Station and Illumina HiSeq™ 2000 System. Sequence tags were prepared using the Illumina Digital Gene Expression Tag Profiling Kit, according to the manufacturer's protocol. Briefly, mRNA was isolated from 6 mg total RNA using magnetic oligo-beads, oligo(dT)s were used as primers to synthesize first and second-strand cDNA, the 5′ ends of the tags were generated by endonuclease *Nla*III, which recognizes and cuts off the CATG sites. The fragments apart from the 3′ cDNA fragments connected to Oligo(dT) beads are washed away and the Illumina adaptor 1 is ligated to the sticky 5′ end of the digested bead-bound cDNA fragments. The junction of Illumina adaptor 1 and the CATG site is the *Mme*I recognition site, an endonuclease with separate recognition sites and digestion sites. *Mme*I cuts 17 bp downstream of the CATG site, producing tags containing adaptor 1. After removal of the 3′ fragments by magnetic bead precipitation, Illumina adaptor 2 was ligated to the 3′ ends of the tags, generating tags with different adaptors at each end to form a tag library. After 15 cycles of linear PCR amplification, 95 bp fragments were purified by 6% TBE PAGE gel electrophoresis, denatured and the single-chain molecules were fixed onto the Illumina Sequencing Chip (flowcell). Each molecule creates a single-molecule cluster sequencing template through *in situ* amplification. Nucleotides labeled with different colors were used to perform sequencing by the sequencing by synthesis (SBS) method; each tunnel can generate millions of raw reads with a sequencing length of 35 bp.

### Sequencing data analysis

The raw data (tag sequences and counts) were submitted to Gene Expression Omnibus (GEO; series GSE38845). Raw data was filtered to remove adaptor tags, low quality tags and tags with a single copy number. Clean tags were classified according to their copy number (as a percentage of the total number of clean tags) and the saturation of the library was analyzed. All CATG +17-nt tags in each gene, not only the most 3′ tag, were taken as reference tags. A preprocessed database of all possible CATG +17-nt tag sequences was created using *Mus musculus* UniGene cluster sets data downloaded from the NCBI (http://www.ncbi.nlm.nih.gov/UniGene/UGOrg.cgi?TAXID=10090, UniGene Build#18?). To monitor mapping events on both strands, both sense and complementary antisense sequences were included. Information on the position of polyadenylation signals was also collected from the transcript database. All clean tags were aligned to the reference sequences, unambiguous tags were annotated and the clean tag number of each gene was counted.

### Determination of gene expression levels and detection of DEGs

A virtual library contains all of the possible CATG +17 base sequences for the reference gene sequences. All clean tags were mapped to the reference sequences (only 1 bp mismatches were considered), filtered and the remainder of the clean tags were designated as unambiguous clean tags. The number of unambiguous clean tags for each gene was calculated and normalized to the number of transcripts per million clean tags (TPM).

To identify DEGs between type B spermatogonia and primary spermatocytes, the number of raw clean tags in each library was normalized to the TPM to obtain the normalized gene expression level. Identification of DEGs was performed as previously described [9] using a false discovery rate (FDR)≤0.001 and a threshold absolute log_2_-fold change ≥1 for the sequence counts across the libraries.

### DEG gene ontology and pathway functional enrichment analysis

Gene ontology (GO), an international standardized gene functional classification system, offers a dynamic-updated controlled vocabulary and strictly defined concept to comprehensively describe the properties of genes and their products in any organism [10], [11]. GO enrichment analysis of functional significance applies a hypergeometric test to map all DEGs to the GO database, looking for significantly enriched GO terms, compared to the genomic background. Pathway analysis was mainly based on the Kyoto Encyclopedia of Genes and Genomes (KEGG) database [12]. Two-sided Fisher's exact tests with multiple testing and the χ^2^ test were used to classify the pathway categories. The FDR was used to correct *P* values; only pathway categories where *P*≤0.05 were chosen. Within significant categories, enrichment (Re) was given by: Re∼nf = n Nf = N, where nf is the number of flagged proteins within the particular category, n is the total number of proteins within the same category, Nf is the number of flagged proteins in the protein reference database list and N is the total number of proteins in the gene reference database list.

### Quantitative real-time PCR analysis

The same independent RNA extractions from GC-1spg and GC-2spd (ts) cells were used for qPCR analysis. The qPCR analysis was performed using the Lightcycler480 (Roche, Basel, Switzerland) with SYBR-Green detection (SYBR PrimeScript RT PCR Kit, TaKaRa) according to the manufacturer's instructions. Each cDNA was analyzed in triplicate, the average threshold cycle (C_t_) was calculated for each sample using the 2^−ΔΔCt^ method, normalized to β-actin and expressed relative to spermatogonia.

### ELISA

GC-1spg and GC-2spd (ts) cells were seeded into a 24-well plate at 8×10^4^ cells/plate. After 48 hours, the medium of the cells in each well were harvested and analyzed for the WNT10A and FGF7 expression level by enzyme-linked immunosorbent assay (ELISA) using mouse WNT10A (CSB-EL026129MO, CUSABIO, China) and FGF7 (EIA-3481, Quantikine, China) ELISA kit following the manufacturer-provided protocol. The Wilcoxon signed rank test was used for comparing paired data. Two-sided *P* values less than 0.05 were considered statistically significant.

### Western blotting

Cultured GC-1spg and GC-2spd (ts) cells were harvested in protein sample buffer. 30 µg of total proteins was loaded in each lane in SDS-PAGE for Western blotting. The antibodies used in Western blotting were RAC (ab13048, Abcam, USA), SFRP2 (12189-1-AP, Proteintech, USA), FGF13 (13201-1-AP, Proteintech,USA) and β-actin (A-4700, Sigma, USA).

## Results

### Analysis of DGE libraries

The major characteristics of the GC-1spg and GC-2spd (ts) tag libraries are summarized in Table 1. Approximately 5.26 million sequence tags were obtained per library, with 386,405 distinct tag sequences. Prior to mapping, the adaptor tags, low quality tags and single copy tags were filtered, producing approximately 4.9 million clean sequence tags per library with 119,914 distinct clean tag sequences (Table 1). The GC-1spg library had higher number of total sequence tags and distinct sequence tags, a higher ratio of distinct tags to total tags and lower percentage of distinct clean high copy number tags than the GC-2spd (ts) library. Saturation analysis indicated that the number of emerging new, distinct tags reduced as the number of total sequence tags increased, when the number of sequencing tags was large enough. When the number of sequenced tags reached 3 million, the library capacity approached saturation (Figure S1-a, b).

**Table 1:** GC-1spg and GC-2spd (ts) DGE library analysis.

	GC-1spg		GC-2spd (ts)	
	Distinct Tag	Total Tag	Distinct Tag	Total Tag
Raw Data	364,298	5,403,320	408,513	5,110,154
Tags Containing N	9,325	21,545	9,200	19,448
Adaptors	16,188	92,497	19,312	102,070
Tag CopyNum<2	217,129	217,129	261,828	261,828
Clean Tag	121,656	5,072,149	118,173	4,726,808
CopyNum> = 2	121,656	5,072,149	118,173	4,726,808
CopyNum>5	45,484	4,861,399	43,294	4,522,513
CopyNum>10	29,703	4,741,945	28,645	4,412,278
CopyNum>20	19,544	4,594,001	19,037	4,271,630
CopyNum>50	11,071	4,322,335	11,046	4,015,259
CopyNum>100	6,695	4,009,298	6,884	3,717,863

Major characteristics of the digital gene expression (DGE) tag libraries generated from mouse GC-1spg and GC-2spd (ts).

### Analysis of tag mapping

A virtual reference tag database, including 78,324 sequences from the *Mus musculus* Unigene database, was generated for tag mapping. All CATG+17 tags in each gene were used as reference tags, not only the most 3′ CATG+17 tag, generating 475,278 reference tag sequences with 446,311 unambiguous tag sequences. The tag sequences were mapped to the reference tag database, allowing one mismatch per alignment to account for polymorphisms. The 66.45% and 66.99% of the distinct clean tags from GC-1spg and GC-2spd (ts) libraries mapped to the Unigene virtual tag database, 51.70% and 52.14% of the distinct clean tags mapped unambiguously and 10.02% and 10.31% of the distinct clean tags did not map to the Unigene virtual tag database, respectively (Table 2). The occurrence of unknown tags is probably due to the incompleteness of mouse genome sequencing data. Most Solexa tags matched to the first or second 3′ CATG site in high-confidence transcripts (Figure S2-a, b).

**Table 2:** Statistics of GC-1spg and GC-2spd (ts) DGE mapping results.

	GC-1spg			GC-2spd (ts)		
Category	DT	TT	Gene	DT	TT	Gene
SG^a^ Tags	56,913	3,926,734	26,604	56,785	3,662,908	26,426
SG^a^ Unambiguous Tags	43,844	2,135,420	16,572	43,863	2,114,111	16,218
ASG^b^ Tags	23,927	439,673	14,531	22,380	392,549	13,922
ASG^b^ Unambiguous Tags	19,051	313,536	9,765	17,747	284,644	9,231
AG^c^ Tags	80,840	4,366,407	28,947	79,165	4,055,457	28,749
AG^c^ Unambiguous Tags	62,895	2,448,956	18,469	61,610	2,398,755	18,081
Unknown Tag	12,187	234,513	0	12,180	211,332	0

Overview of tags mapping to the Unigene database in the digital gene expression (DGE) tag libraries generated from mouse GC-1spg and GC-2spd (ts).

a Sense Gene,

b Anti-Sense Gene,

c Sense & Anti-Senese.

To analyze the depth of transcriptome sampling, the rate of increase in the number of genes (sense+antisense strand) identified as the size of the libraries increased was studied. When the library size reached two million tags, the number of identified expressed genes did not increase for either total or unambiguous tags (Figure S1-a, b), demonstrating that the capacity of the libraries approached saturation.

### Detection of differentially expressed genes by DGE and validation by qPCR

**Figure 1:**
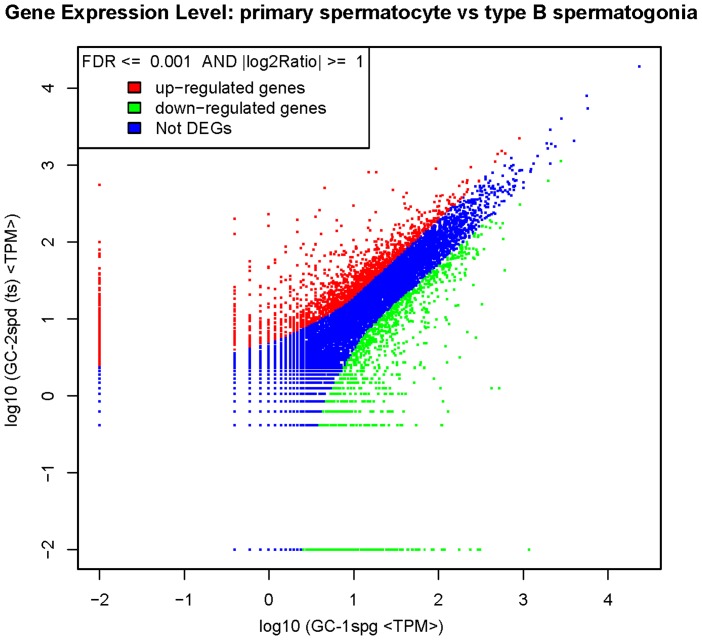
Scatter plot indicates the comparing results of log transformed gene expression levels and differentially expressed genes' distribution between GC-1spg and GC-2spd (ts).

**Figure 2:**
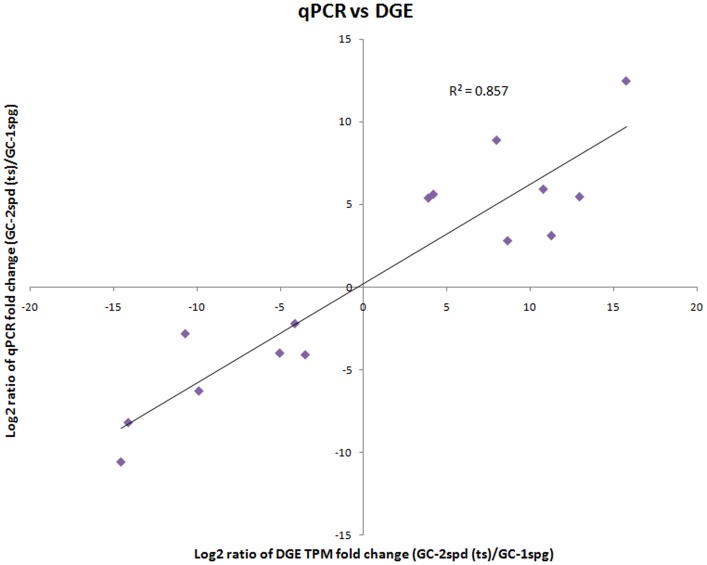
Correlation between the expression fold change level of 15 randomly selected differently expressed genes between GC-1spg and GC-2spd (ts). The selected genes cover different expression levels of both up- and down-regulated genes defined by digital gene expression, and the fold change level validated by qPCR. Gene expression levels are presented at the normalized gene expression levels in primary spermatocytes, relative to type B spermatogonia.

A total of 2,823 genes were identified as DEGs during the transformation from type B spermatogonia to primary spermatocytes; 1,651 genes were up-regulated and 1,172 genes were down-regulated in primary spermatocytes compared to type B spermatogonia (Figure 1; Table S1). Some genes are significantly differentially expressed between the two kinds of cell type, such as *Sfrp2, Hspa1b, Wnt10a*, *Fgf5* and so on, which look like transcriptional switch on and off (Table S2). To validate if DGE results are reliable, we have done qPCR analysis for 15 representative differentially expressed genes of different expression levels. Solexa sequencing indicated that *Sfrp2, Hspa1b, Notch1, Pdgfrb, Prickle3, Fgf7, Ccl5* and *Cntln* were up-regulated and *Baiap2l1, Itgb7, Cxcl1, Fgf13, Bcl2l15, Wnt10a, Fgf5* were down-regulated in GC-2spd (ts) compared with GC-1spg. The qPCR results (Table S2) indicated that the expression fold change detected by DGE and qPCR was highly correlated (r^2^ = 0.857) (Figure 2). The qPCR validation results confirmed the accuracy and reliability of the expression changes detected by DGE analysis, which means that we can make reasonable deduction from the DEGs functional enrichment analysis.

### Functional enrichment analysis of differentially expressed genes

**Figure 3:**
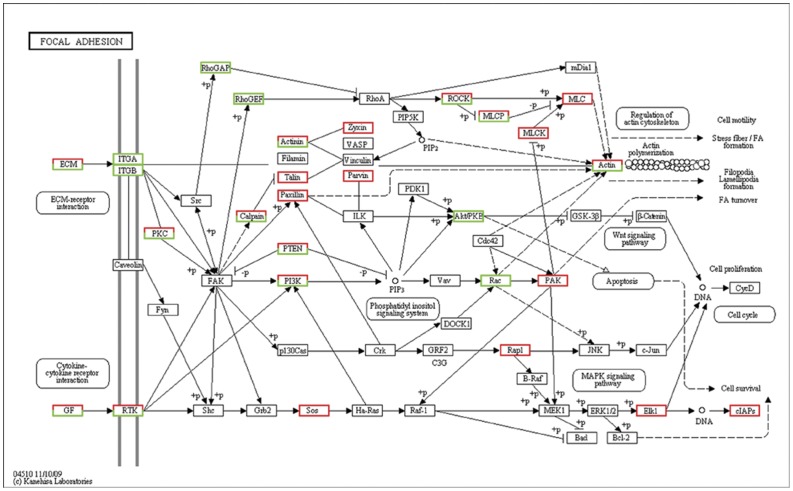
Differentially expressed genes involved in the focal adhesion signaling pathway during early spermatogenesis. Genes up-regulated and down-regulated during the development process from GC-1spg to GC-2spd (ts) are marked in red and green, respectively.

**Figure 4:**
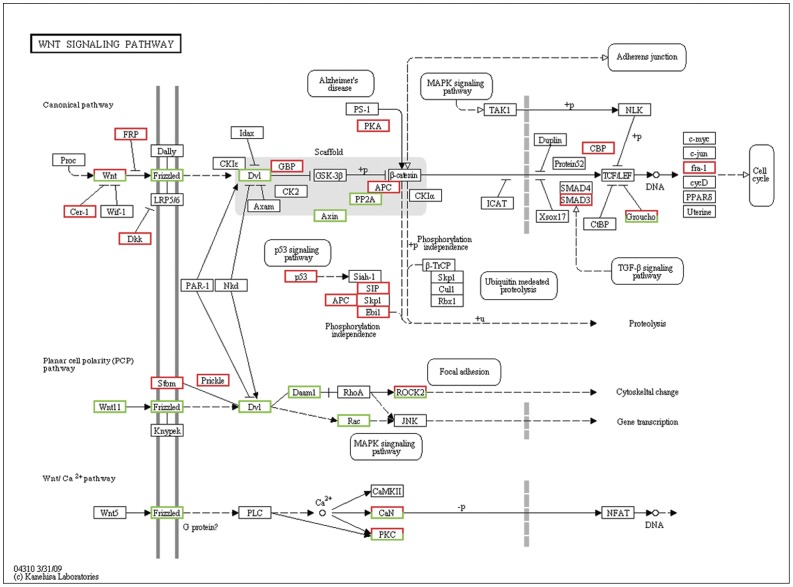
Differentially expressed genes involved in Wnt signaling pathway during early spermatogenesis. Genes up-regulated and down-regulated during the development process from GC-1spg to GC-2spd (ts) are marked in red and green, respectively.

**Figure 5:**
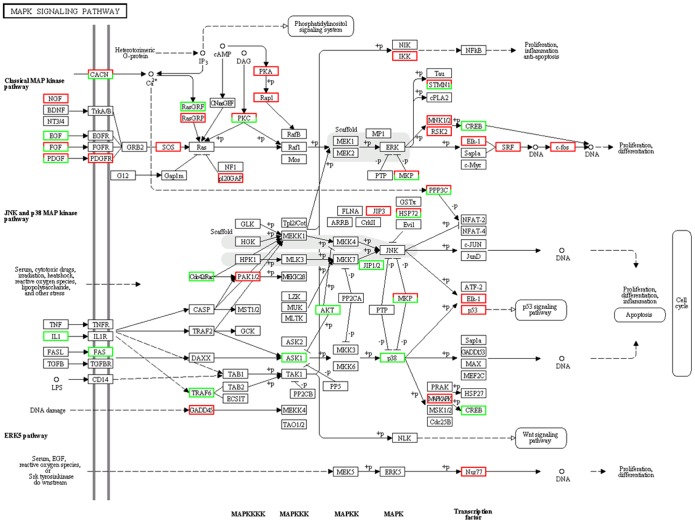
Differentially expressed genes involved in MAPK signaling pathway during early spermatogenesis. Genes up-regulated and down-regulated during the development process from GC-1spg to GC-2spd (ts) are marked in red and green, respectively.

**Figure 6:**
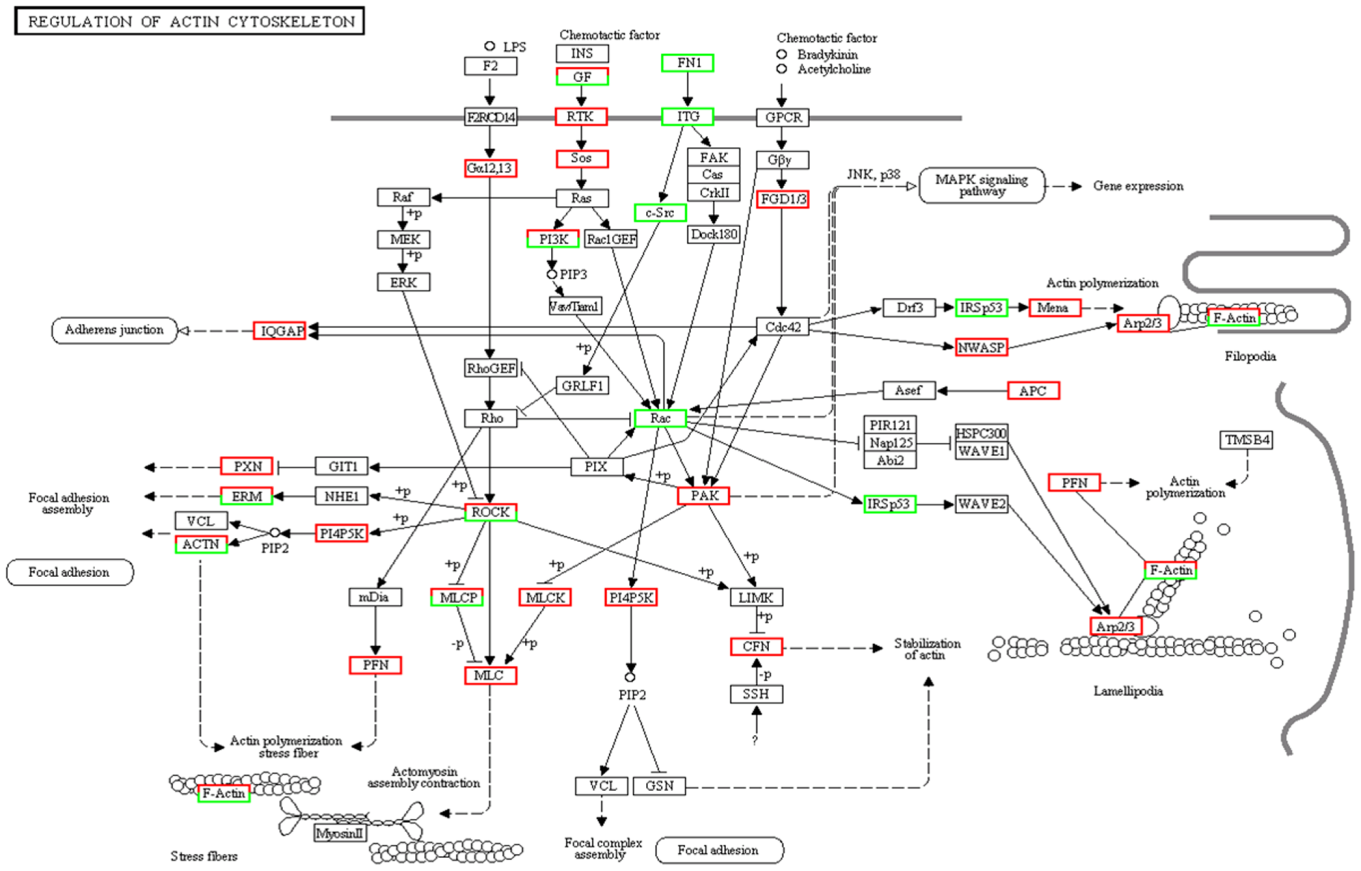
Differentially expressed genes involved in the regulation of action cytoskeleton pathway during early spermatogenesis. Genes up-regulated and down-regulated during the development process from GC-1spg to GC-2spd (ts) are marked in red and green, respectively.

GO and pathway analysis were performed to characterize the function of the DEGs associated with the transition from type B spermatogonia to primary spermatocytes based on the annotation data of GO and KEGG databases using two-sided Fisher's exact tests. A variety of signaling pathways including regulation of focal adhesions dynamic (Figure 3), Wnt signaling transduction (Figure 4), MAPK signaling transduction (Figure 5), actin cytoskeleton dynamic (Figure 6), cytokine-cytokine receptor interactions, tight junctions and adherens junctions were significantly affected (Table 3, Figure S3). Relationships were observed between different enriched pathways associated with the transition from GC-1spg to GC-2spd (ts). The down-regulated gene *Rac* is involved in 8 of 23 enriched pathways (Table 3), which means that it may be the regulatory node of the differentiation process from GC-1spg to GC-2spd (ts). Focal adhesion pathway (Figure 3, Table S1) was the most significantly enriched (Table 3), and Wnt related signaling pathway which can be divided into the canonical pathway and non-canonical pathway was also associated with the transition (Figure 4).

**Table 3:** Functional enriched pathways of differentially expressed genes.

Enriched pathway	P-value	Q-value	*Rac* *
Focal adhesion	1.39E-10	2.81E-08	Yes
Pathways in cancer	2.53E-09	2.56E-07	Yes
Regulation of actin cytoskeleton	7.53E-06	4.33E-04	Yes
Axon guidance	8.69E-06	4.33E-04	Yes
Hedgehog signaling pathway	1.07E-05	4.33E-04	No
p53 signaling pathway	1.43E-05	4.82E-04	No
ECM-receptor interaction	2.72E-05	7.85E-04	No
Apoptosis	4.02E-05	1.02E-03	No
Small cell lung cancer	8.93E-05	2.00E-03	No
Wnt signaling pathway	0.000167814	3.39E-03	Yes
Chemokine signaling pathway	0.000184758	3.39E-03	No
Melanoma	0.000373506	5.85E-03	No
Basal cell carcinoma	0.000376317	5.85E-03	No
Notch signaling pathway	0.000886656	1.28E-02	No
Dorso-ventral axis formation	0.001329788	1.79E-02	No
Cytokine-cytokine receptor interaction	0.001977997	2.42E-02	No
Lysosome	0.00203415	2.42E-02	No
Pancreatic cancer	0.00265948	2.96E-02	Yes
Tight junction	0.002786753	2.96E-02	No
Melanogenesis	0.003778263	3.68E-02	No
MAPK signaling pathway	0.003923015	3.68E-02	Yes
Adherens junction	0.004009564	3.68E-02	Yes
Prion diseases	0.005680357	4.99E-02	No

Significantly enriched signaling pathways for the differentially expressed genes in the digital gene expression (DGE) tag libraries generated from mouse GC-1spg and GC-2spd (ts).

* If Rac is involved in the pathway.

### Detection of protein expression level of candidate marker genes

**Figure 7:**
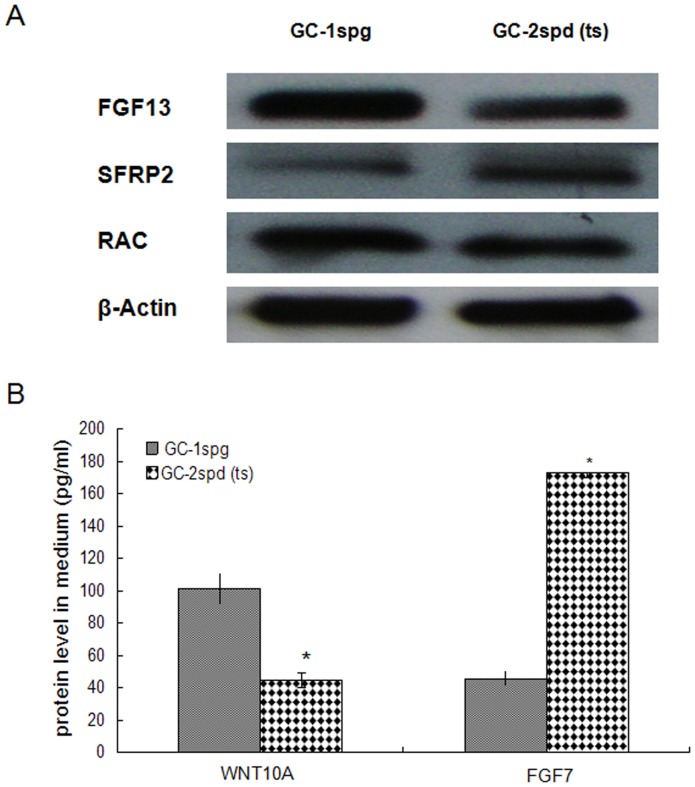
Protein levels of FGF13, SFRP2, RAC, WNT10A and FGF7. (A) Protein levels of FGF13, SFRP2 and RAC in GC-1spg and GC-2spd (ts) cells were detected by western blotting respectively. β-Actin were used to normalize the individual expression levels. (B) Protein level of WNT10A and FGF7 in the medium supernatants from GC-1spg and GC-2spd (ts) cells were determined by ELISA. The data are presented as the mean plus SEM from a representative of three independent experiments, with each performed in triplicate. Statistically significant differences between GC-1spg and GC-2spd (ts) cells are indicated above the bars: * means P<0.05.

According to the results of DGE, we chose several vital genes which may play crucial role in regulating or modulating focal adhesion pathway, Wnt signaling pathway, regulation of actin cytoskeleton and MAPK signaling pathway to detect the protein expression levels. Among them, SFRP2, FGF13 and RAC were detected by Western blotting, and WNT10A & FGF7 by ELISA. Western blotting analysis confirmed weakly higher protein levels of RAC, significantly higher level of FGF13 and lower level of SFRP2 in GC-1spg cell than GC-2spd (ts) cell, which were consistent with the RNA level difference detected by DGE (Figure 7A, Table S2). ELISA analysis confirmed the higher protein expression levels of WNT10A and lower levels of FGF7 in GC-1spg (*p*<0.05), which were also consistent with the RNA level detected by DGE and qPCR (Figure 7B).

## Discussion

The basement membrane and Sertoli cells which line the seminiferous epithelium of adult mammalian testes are in close contact with germ cells at different stages of development [13]. It plays a significant role in regulating the process of spermatogenesis and maintaining the integrity of the blood-testis barrier (BTB) [14], [15]. Recent studies have elucidated the pivotal role of the basement membrane in supporting Sertoli cell and germ cell function in the seminiferous epithelium [16]. Preleptotene spermatocytes differentiated from type B spermatogonia residing at the basal compartment must traverse the blood-testis barrier (BTB) to enter the adluminal compartment to prepare for meiosis [17], so cell movement is indispensable to spermatogenesis. In this study, DEGs functional enrichment analysis shows that the mainly enriched pathways during the early spermatogenesis are strongly related to the dynamic of junction or adhesion such as focal adhesions and regulation of actin cytoskeleton (Figure 3,6), and these signals are translocated to the nucleus where they phosphorylate transcription factors, thereby regulating Wnt- and MAPK-target genes (Figure 4,5).

### Variation of biological pathways during the early spermatogenesis

The junction status and shape of spermatogonia and spermatocytes are significantly different. The translocation of germ cells across the seminiferous epithelium during spermatogenesis requires extensive restructuring of the cell junctions at the Sertoli-germ interface [4], [18]. It is understandable that focal adhesion is the most significantly enriched (Table 3). Focal adhesion can sense ECM, cell surface, physiological stress and mechanical stress through integrin, caveolin and RTK [19], [20], [21]. The responses of this pathway include cell growth or death, cell motility, cytoskeleton reorganization and gene expression [21]. In our study, the down-regulated *ITGA* and *ITGB* in GC-2spd (ts) in combination with the down-regulated *RhoGAP*, *RhoGEF*, *Rac* and up-regulated *MLC* and *PAK* finally lead to the regulation of actin cytoskeleton. Once the dynamic of actin cytoskeleton change, the cell will migrate and move towards the adluminal compartment to prepare for meiosis.

Wnt/Frizzled signaling pathway controls developmental, physiological, and pathological processes. This aspect of Wnt signaling is comprised of a number of signaling branches that are subsequently integrated into the regulation of actin cytoskeleton during cell polarization and cell migration [22], [23]. Rho family of small GTPases (Rac and Rho) are required for non-canonical Wnt signaling pathway [24], which was affected during the transition from type B spermatogonia to primary spermatocytes.

MAPK signaling pathway is a basic signal transduction pathway that couples intracellular responses to the binding of growth factors to cell surface receptors, which regulates cell proliferation, survival, differentiation and apoptosis [25]. MAPK signaling pathway is composed of three different major signaling pathways—classical MAP kinase pathway, JNK and p38 MAP kinase pathway and ERK5 pathway [26]. In our studies, the classical MAP kinase pathway was activated by some up- or down- regulated *FGFs* such as *FGF13*, *FGF5* and *FGF7*. The regulation of *Ras* family genes and *PKA* of MAPK downstream signaling pathway will finally lead to the up-regulation of c-Fos in GC-2spd (ts) (Figure 5). In combination with c-Jun which is activated through double phosphorylation by the JNK pathway [27], the two active proteins will form the AP-1 early response transcription factor which can regulate gene expression in response to a variety of stimuli, including cytokines and growth factors [28] and control a number of cellular processes including differentiation, proliferation and apoptosis [29].

Regulation of the dynamic actin cytoskeleton includes signaling to the cytoskeleton through GPCRs, integrins and receptor tyrosine kinases (RTKs) which can lead to diverse effects on the cells activity such as changes in cell shape, migration and proliferation [30]. Dynamic actin coordinated with other signaling pathways such as MAPK and Wnt signaling pathway is required for most cellular actin-dependent processes, Regulation of dynamic actin can result in actin assembly or disassembly at the focal adhesion (Figure 6). Intracellular regulation of the cell's response to external cues occurs through a large number of signaling cascades that include the Rho family of small GTPases (*Rho*, *Rac* and *Cdc42*) and their activators, guanine nucleotide exchange factors (*GEFs*), their downstream protein kinase effectors, including Rho-kinase/ROCK and p21 activated kinase (*PAK*), as well as through direct binding of the GTPases to several actin regulatory proteins [30]. These cascades converge on proteins that directly regulate the behavior and organization of the actin cytoskeleton. It is believed that the transcriptional and post-transcriptional regulation of the genes involved in the regulation of the assembly and disassembly of cell junctions will result in the progressive movement of germ cells from the basal compartment to the adluminal space in order to complete spermatogenesis [31], [32].

Based on the analysis, all these enriched pathways in fact interact with each other, they work as an integrated regulatory network which will finally change global gene expression pattern of type B spermatogonia to promote cell migration, dynamic of junctions and differentiation. Therefore, it is crucial that the transcriptional regulations of junction proteins should be fully characterized so that we can completely understand spermatogenesis.

### *Rac* is a key regulatory node during early spermatogenesis

*Rac* gene is a member of the *Rho* superfamily of GTPases [33], which are known to regulate many cellular processes including cell movement and cell adhesion and implicated in the spatial-tempo control of cytoskeletal actin filament organization and adhesion dynamic regulation [34], [35], [36]. Cell movement and dynamic of cell adhesion are crucial to spermatogenesis. It is undoubted that *Rac* plays a key role during spermatogenesis [37]. In our studies, *Rac* GTPase is down regulated in GC-2spd (ts) and affects focal adhesion pathway, Wnt/frizzled non-canonical signaling pathway and 6 other enriched pathways as an interlinked gene (Figure 3, 4, 5, 6, Table 3). *Rac* regulates the dynamic of actin cytoskeleton which in turn control changes in cell shape, and plays important roles in MAPK signaling pathway which ultimately influences gene expression. The interplay among Wnt non-canonical signaling pathway, adhesion and cytoskeletal dynamic observed in the development process from type B spermatogonia to primary spermatocytes from this study provides us new clues on the regulation of spermatogenesis. Wnt signaling pathway may play a central role in the regulation of spermatogenesis by down-regulating or inhibiting the expression of *Rac* on both RNA and protein levels (Figure 7A). Although the physiological significance of *Rho* GTPases especially *Rac* in spermatogenesis remains largely unexplored, the coordinated influence on corresponding signaling pathway and the interaction between each pathway linked by *Rac* will give us more insights on the central regulatory role of *Rac* during spermatogenesis.

### Candidate markers which can discriminate GC-1spg from GC-2spd (ts)

Our findings indicate that some key regulatory genes can be recognized as marker genes because they can discriminate primary spermatocyte from type B spermatogonium (Table S1). These marker genes function as the key regulators or mediators which mainly regulate adhesion dynamic and cytoskeleton dynamic. Among the DEGs detected by Sloxa sequencing and confirmed by qPCR, *Sfrp2, Wnt10a*, *Baiap2l1* and *Fgf13* were defined as candidate marker genes. These genes also have similar expression pattern in rat spermatogonia and spermatocytes (data not shown).

***Sfrp2***-Secreted frizzled-related proteins act as Wnt signaling antagonists and are implicated in a variety of biological processes, *Sfrp1* and *Sfrp2* are required for normal mouse male sexual development [38]. *Sfrp2* is expressed during development of a variety of tissues [39]. Secreted frizzled-related protein 1 (sFRP1) was reported to regulate spermatid adhesion in the testis via dephosphorylation of focal adhesion kinase and the nectin-3 adhesion protein complex [40]. The significant differential expression of *Sfrp2* between GC-1spg and GC-2spd (ts) (Figure 7A) indicates that *Sfrp2* may play a vital role in early germ cell differentiation by antagonizing Wnt signaling pathway to promote differentiation and cell movement, which induces the transition from spermatogonia progeny to primary spermatocyte.

***Wnt10a*** is a member of the *Wnt* gene family, which consists of structurally-related genes encoding secreted signaling proteins implicated in oncogenesis and several developmental processes, including the regulation of cell fate and patterning during embryogenesis [41], [42]. Traditionally, it is assumed that Wnt proteins act as stem cell growth factors to promote the maintenance and proliferation of stem cells [43]. Lower expression level of *Wnt10a* and higher level of *Sfrp2* in GC-2spd (ts) concordantly inhibit Wnt signaling pathway which promotes germ cell differentiation.

***Baiap2l1*** encodes a member of the IRSp53/MIM homology domain (IMD) family and is involved in the signal transduction pathways which link deformation of the plasma membrane and remodeling of the actin cytoskeleton. *Baiap2l1* promotes actin assembly and membrane protrusions when overexpressed in mammalian cells, and is essential for formation of potent actin assembly complexes [44]. We observed that *Baiap2l1* was expressed at higher level in GC-1spg than GC-2spd (ts), which strongly suggests that *Baiap2l1* might be related to spermatogonia developmental process induced by the basement membrane, a modified form of extracellular matrix in the testis.

***Fgf13*** is a member of the FGF family, which possess broad mitogenic and cell survival activities and is involved in a variety of biological processes including embryonic development, cell growth, morphogenesis, tissue repair, tumor growth and invasion [45]. Cory *et al.* reported that expression of *Fgf13* could be detected within the testis cords of the male genital ridge by *in situ* hybridization by embryonic day (E) 13.5, suggesting that *Fgf13* might contribute to signal transduction within the pre-Sertoli cells of the developing male genital ridge [46]. Our study indicates that *Fgf13* may exert similar function to *Baiap2l1*, and plays an important role in the interaction between Sertoli cells and germ cells.

## Conclusions

This transcriptome profiling study delineates global gene expression patterns in both mouse type B spermatogonia and primary spermatocytes. Functional enrichment analysis of differentially expressed genes between the two types of cells shows that the basic molecular mechanism of early spermatogenesis is strongly related to the dynamic of cell junction and regulation of cytoskeleton. In these pathways, the signals of internal or external dynamic changes will ultimately be transmitted to the nuclear to change expression patterns of key genes that can promote cell differentiation. The information of this study provides a solid foundation for further characterization of the gene regulatory process during the early differentiation of male germ cells. Further biochemical and physiological study for the candidate marker genes will be implemented in the future.

## Supporting Information

Table S1 **Summary of the differentially expressed genes (DEGs) identified in the digital gene expression tag libraries generated from GC-1spg and GC-2spd (ts).**
(XLS)

Table S2 **Location of the differentially expressed genes (DEGs) within the 23 significantly enriched KEGG pathways in the digital gene expression tag libraries generated from GC-1spg and GC-2spd (ts).**
(XLS)

Figure S1 **Saturation of the digital gene expression (DGE) tag libraries generated from GC-1spg (A) and GC-2spd (ts) (B).** The effect of library size on the number of genes identified was analyzed; the rate of increase for all identified genes and all genes identified by unambiguous tags declined as the library size increased.
(DOC)

Figure S2 **Positions of tags in the digital gene expression (DGE) tag libraries generated from GC-1spg (A) and GC-2spd (ts) (B).** Ideally the tag was the most 3′ tag; however, the tags may also be the second or third most 3′ tag due to alternative splicing or incomplete enzyme digestion.
(DOC)

Figure S3 **Significantly enriched signaling pathways of DEGs detected between GC-1spg and GC-2spd (ts).** P values<0.05 and a FDR of 0.05 were selected as significant criteria for the two-sided Fisher's exact test.
(DOC)
