# Supplementary material for: Digital Gene Expression Tag Profiling Analysis of the Gene Expression Patterns Regulating the Early Stage of Mouse Spermatogenesis
Source: PLoS One. 2013 Mar 15;8(3):e58680. doi: 10.1371/journal.pone.0058680 (PMC3598852; doi:10.1371/journal.pone.0058680)
Supplement: Figure S1 — Saturation of the digital gene expression (DGE) tag libraries generated from GC-1spg (A) and GC-2spd (ts) (B). The effect of library size on the number of genes identified was analyzed; the rate of increase for all identified genes and all genes identified by unambiguous tags declined as the library size increased. (DOC) [file pone.0058680.s003.doc]

(A) Spermatogonia (B) Spermatocytes


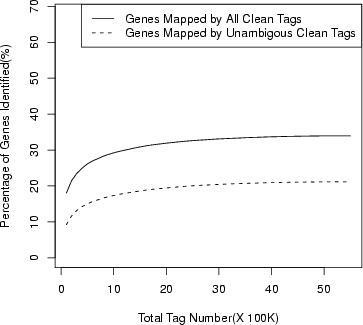

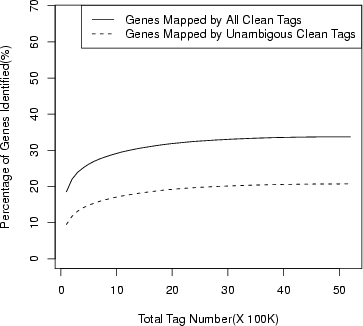


**Figure S1. Saturation of the digital gene expression (DGE) tag libraries generated from GC-1spg (A) and GC-2spd (ts) (B).** The effect of library size on the number of genes identified was analyzed; the rate of increase for all identified genes and all genes identified by unambiguous tags declined as the library size increased.
