# Supplementary material for: Digital Gene Expression Tag Profiling Analysis of the Gene Expression Patterns Regulating the Early Stage of Mouse Spermatogenesis
Source: PLoS One. 2013 Mar 15;8(3):e58680. doi: 10.1371/journal.pone.0058680 (PMC3598852; doi:10.1371/journal.pone.0058680)
Supplement: Figure S2 — Positions of tags in the digital gene expression (DGE) tag libraries generated from GC-1spg (A) and GC-2spd (ts) (B). Ideally the tag was the most 3′ tag; however, the tags may also be the second or third most 3′ tag due to alternative splicing or incomplete enzyme digestion. (DOC) [file pone.0058680.s004.doc]

(A) Spermatogonia (B) Spermatocytes


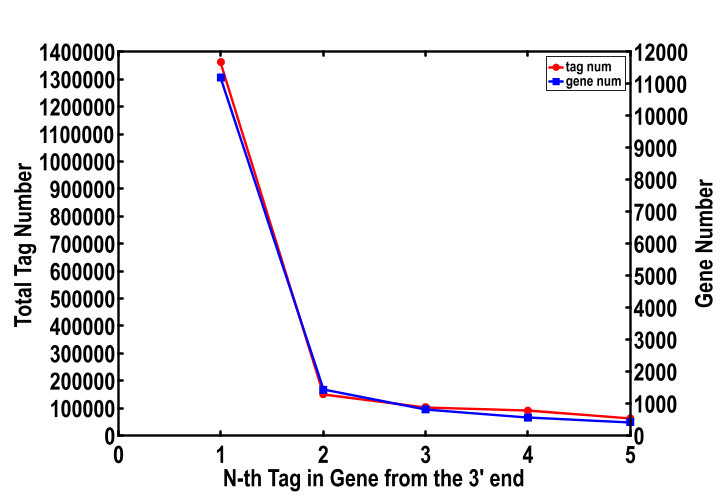

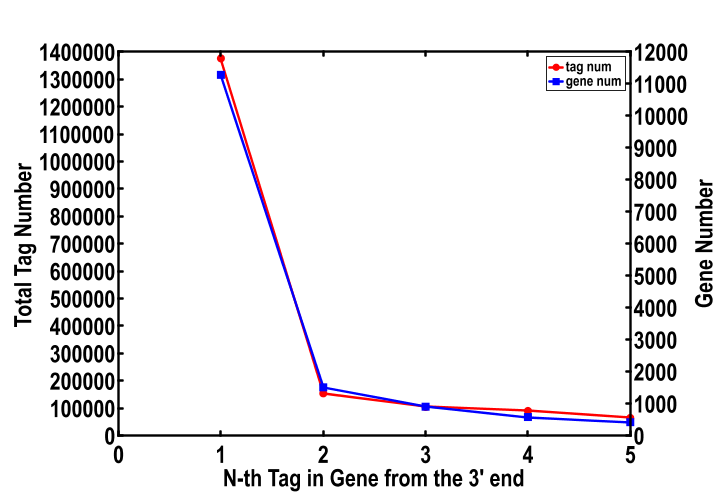


**Figure S2. Positions of tags in the digital gene expression (DGE) tag libraries generated from GC-1spg (A) and GC-2spd (ts**) (B). Ideally the tag was the most 3` tag; however, the tags may also be the second or third most 3` tag due to alternative splicing or incomplete enzyme digestion.
