# Supplementary material for: Digital Gene Expression Tag Profiling Analysis of the Gene Expression Patterns Regulating the Early Stage of Mouse Spermatogenesis
Source: PLoS One. 2013 Mar 15;8(3):e58680. doi: 10.1371/journal.pone.0058680 (PMC3598852; doi:10.1371/journal.pone.0058680)
Supplement: Figure S3 — Significantly enriched signaling pathways of DEGs detected between GC-1spg and GC-2spd (ts). P values<0.05 and a FDR of 0.05 were selected as significant criteria for the two-sided Fisher's exact test. (DOC) [file pone.0058680.s005.doc]

**(A)**


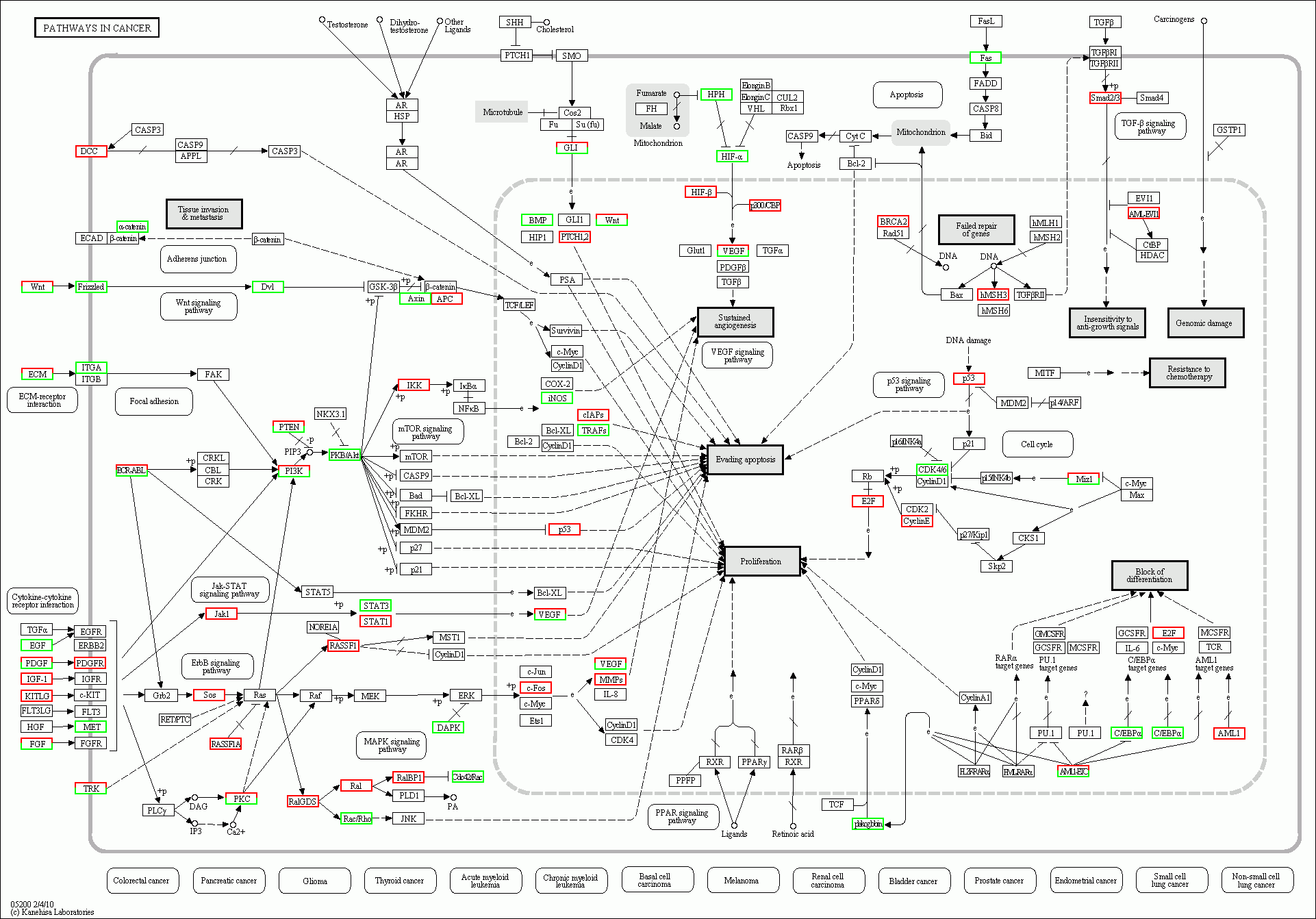


KEGG map05200: Pathway in cancer

(B)
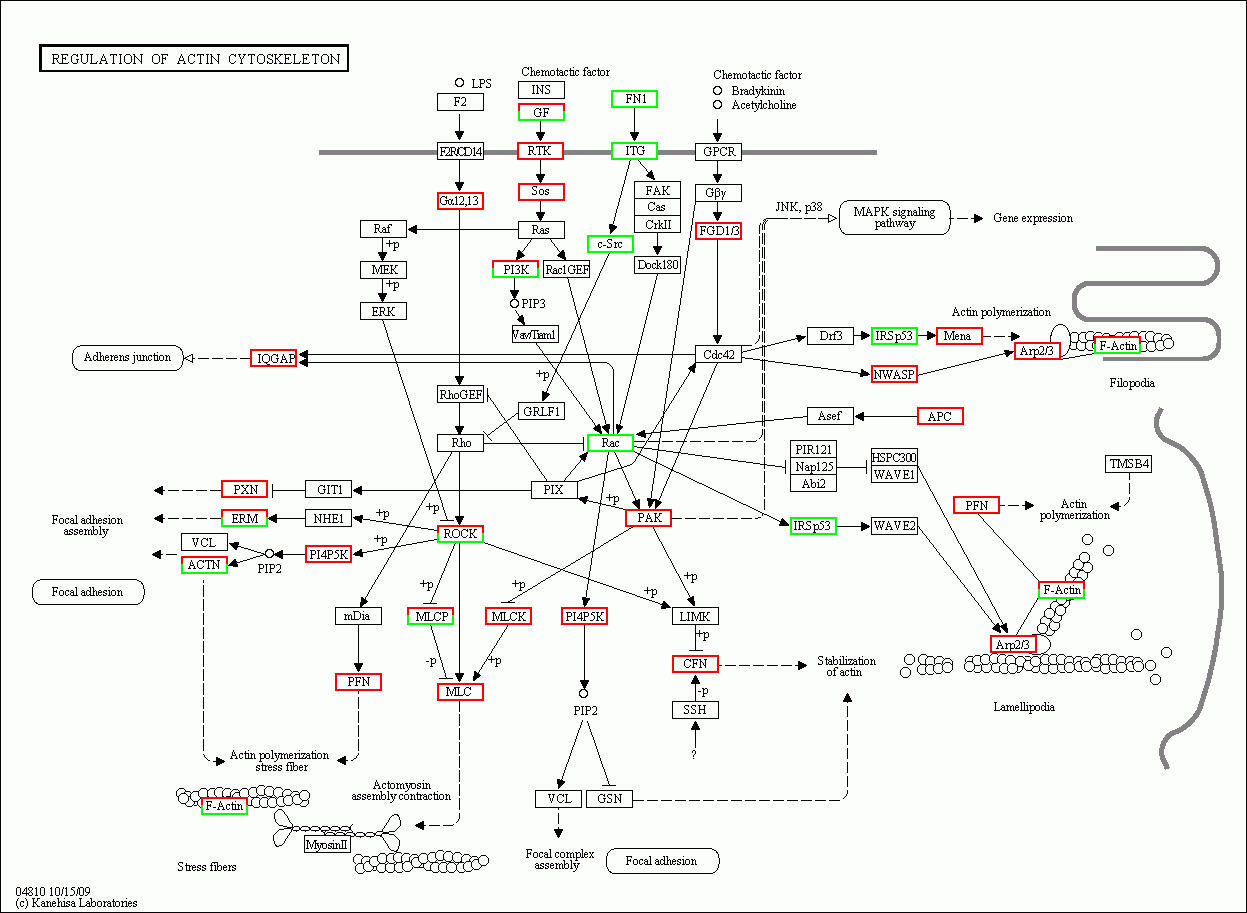


KEGG map04810: Regulation of actin cytoskeleton

(C)


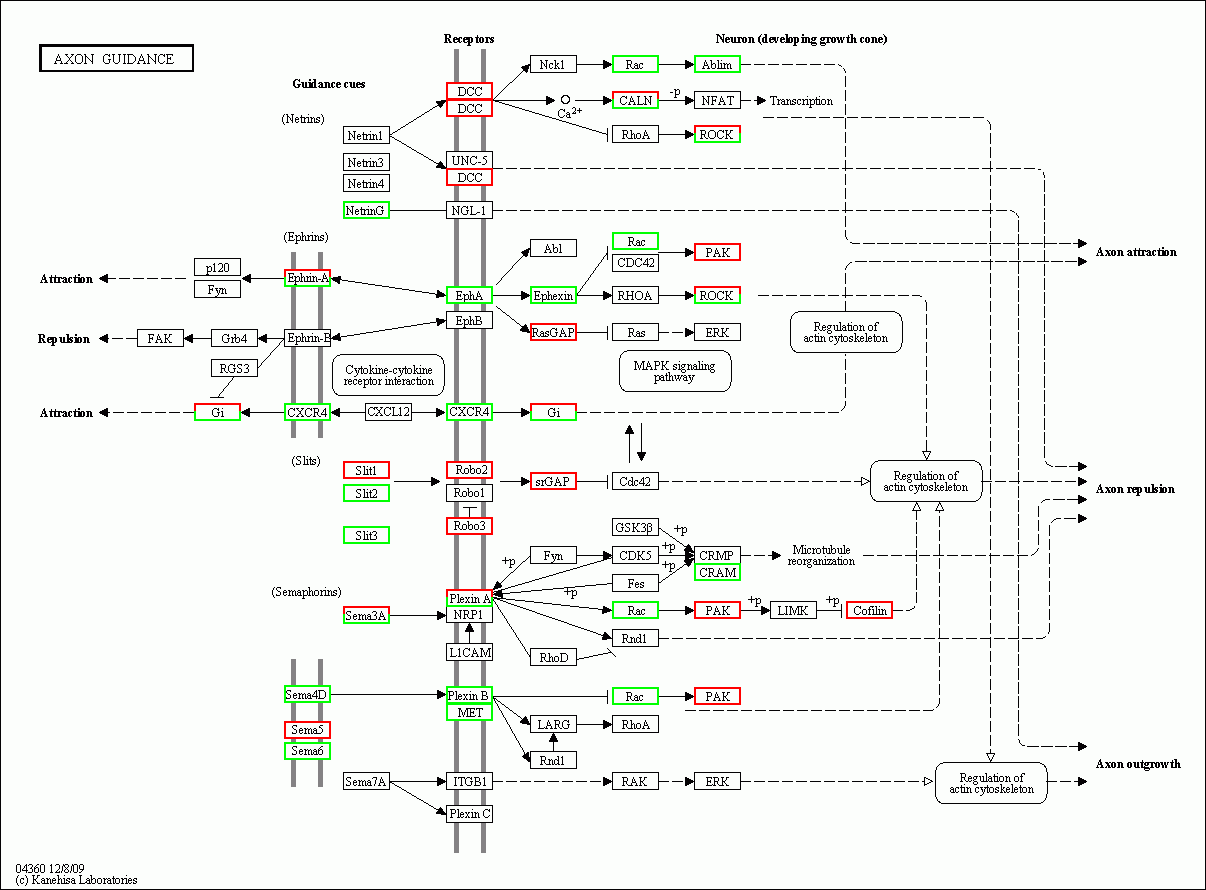


KEGG map04360: Axon guidance

(D)


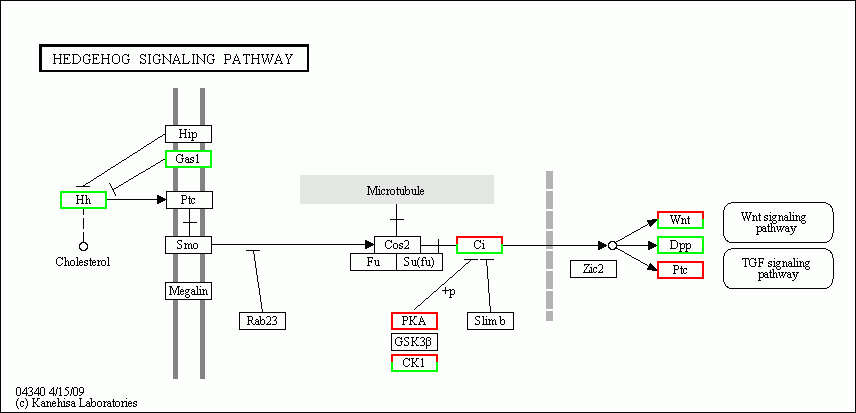


KEGG map04340: Hedgehog signaling pathway

(E)


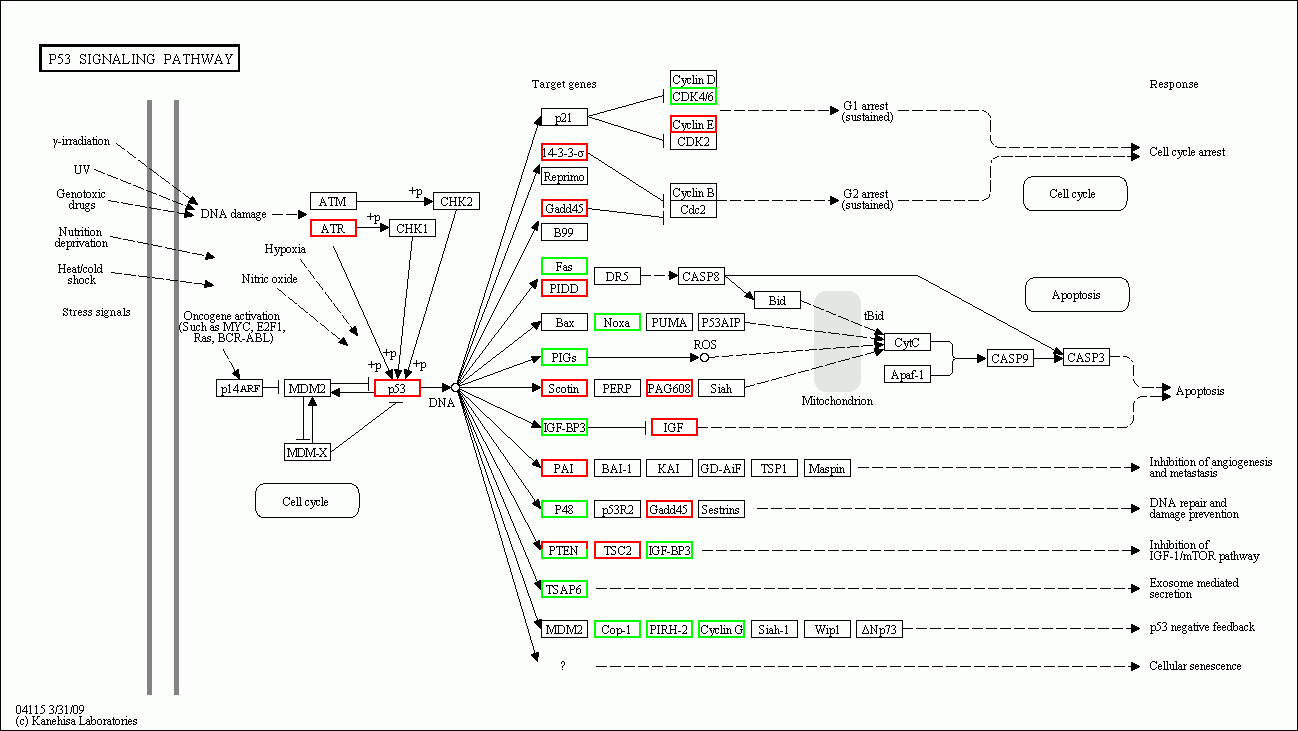


KEGG map04115: p53 signaling pathway

(F)

**
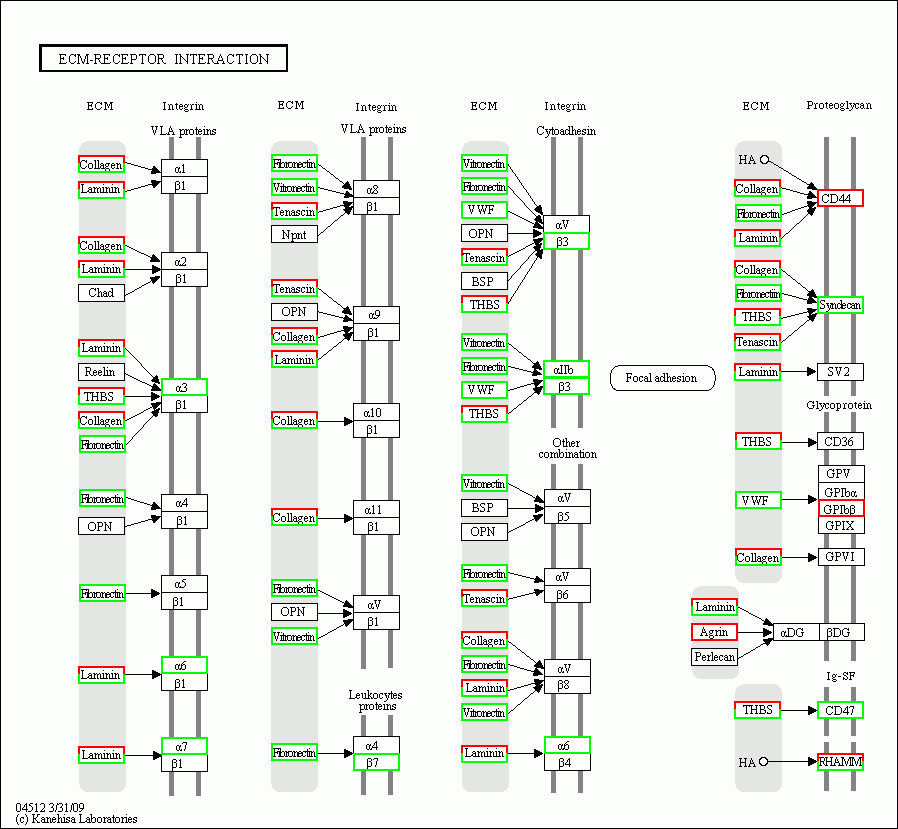
**

KEGG map04512: ECM-receptor interaction

(G)


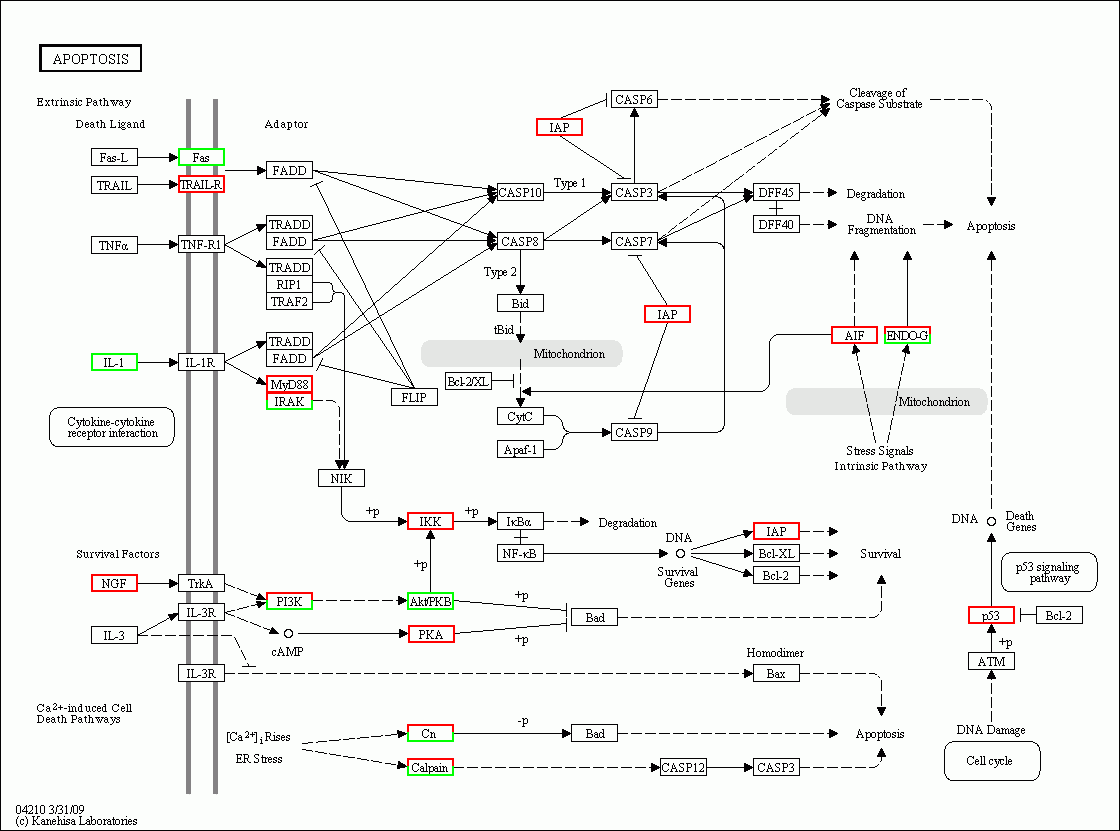


KEGG map04210: Apoptosis

(H)


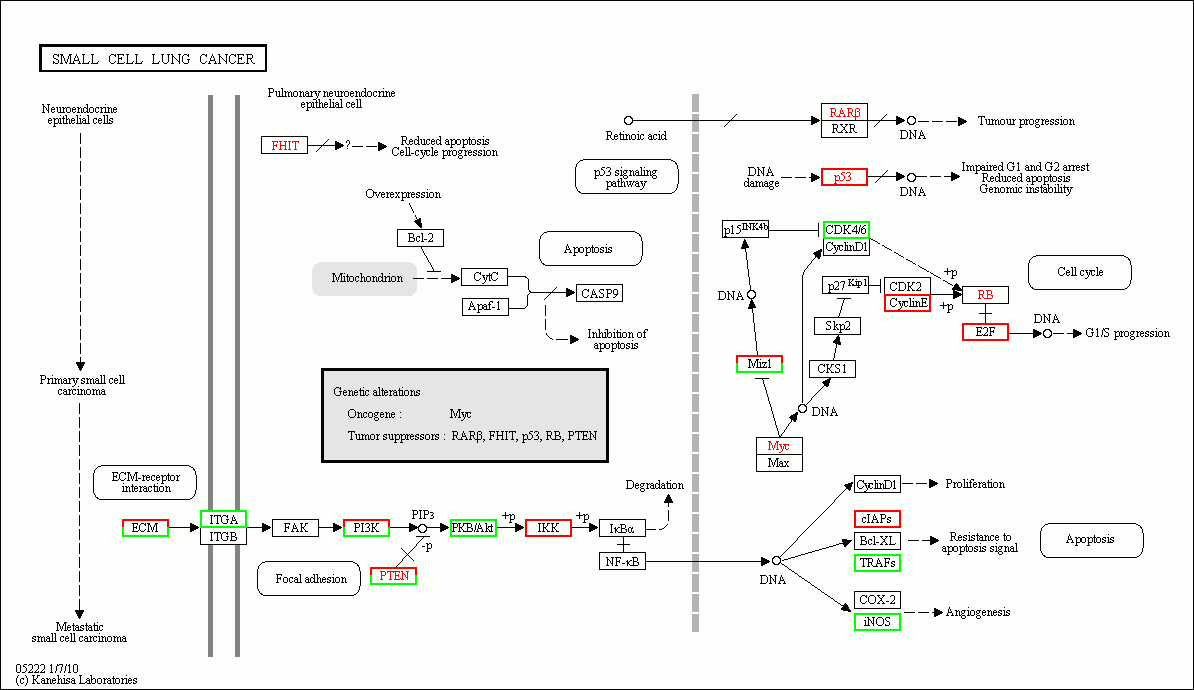


KEGG map05222: Small cell lung cancer

(I)


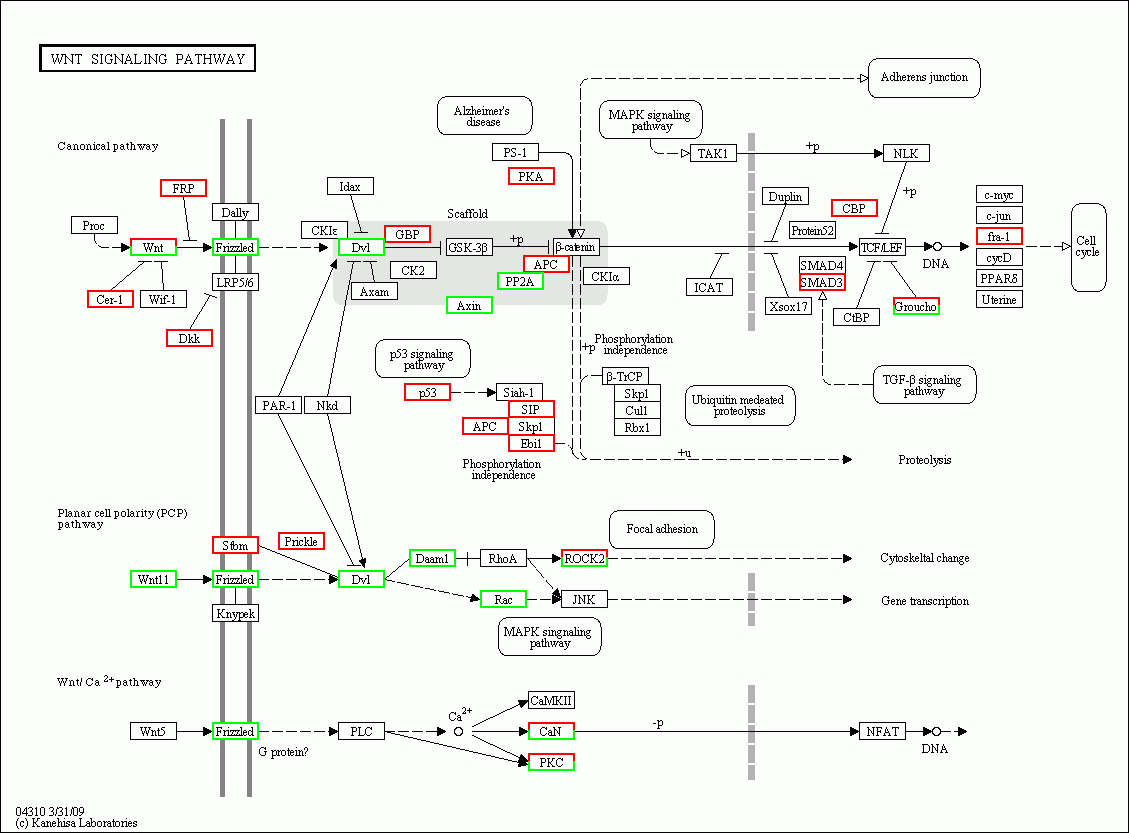


KEGG map04310: WNT signaling pathway

(J)


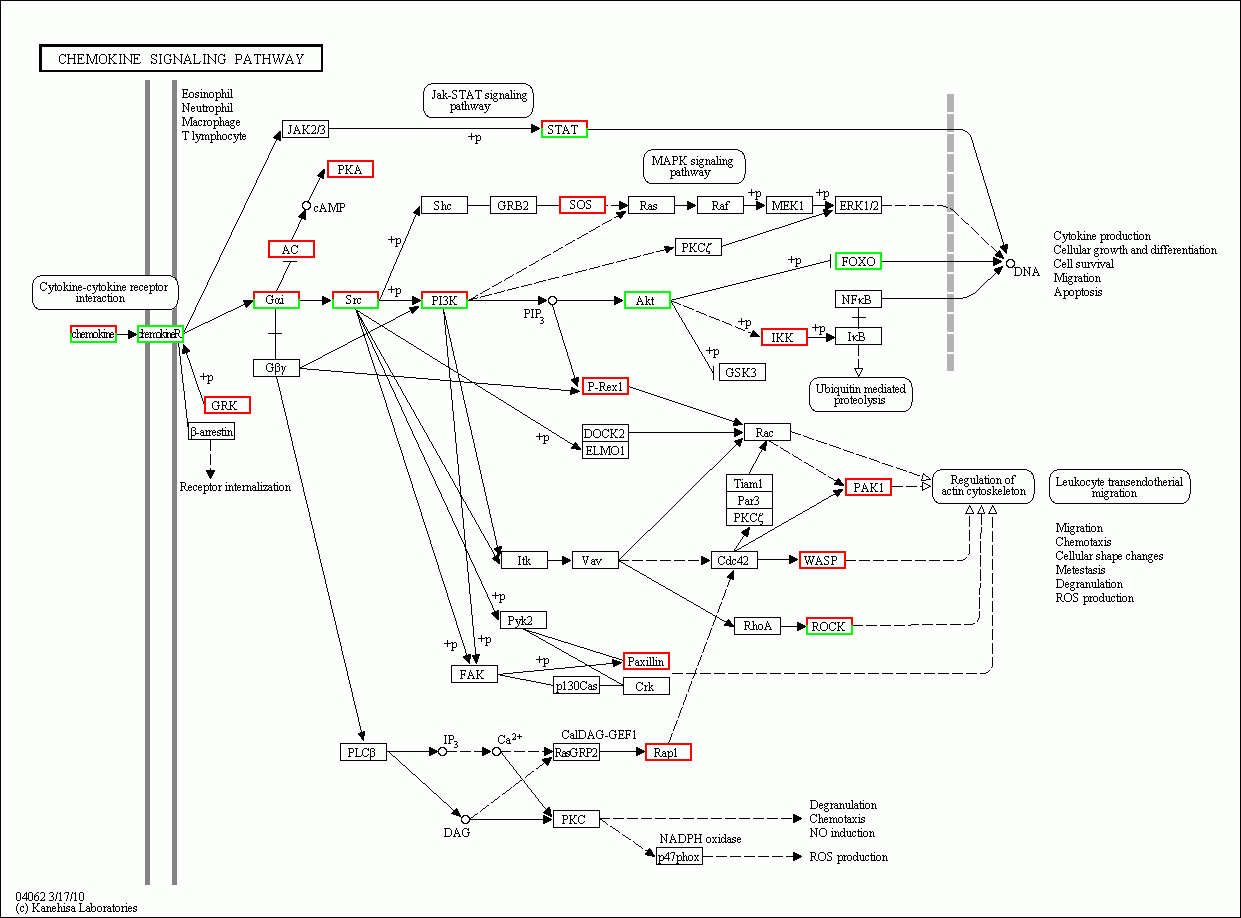


KEGG map04062: Chemokine signaling pathway

(K)


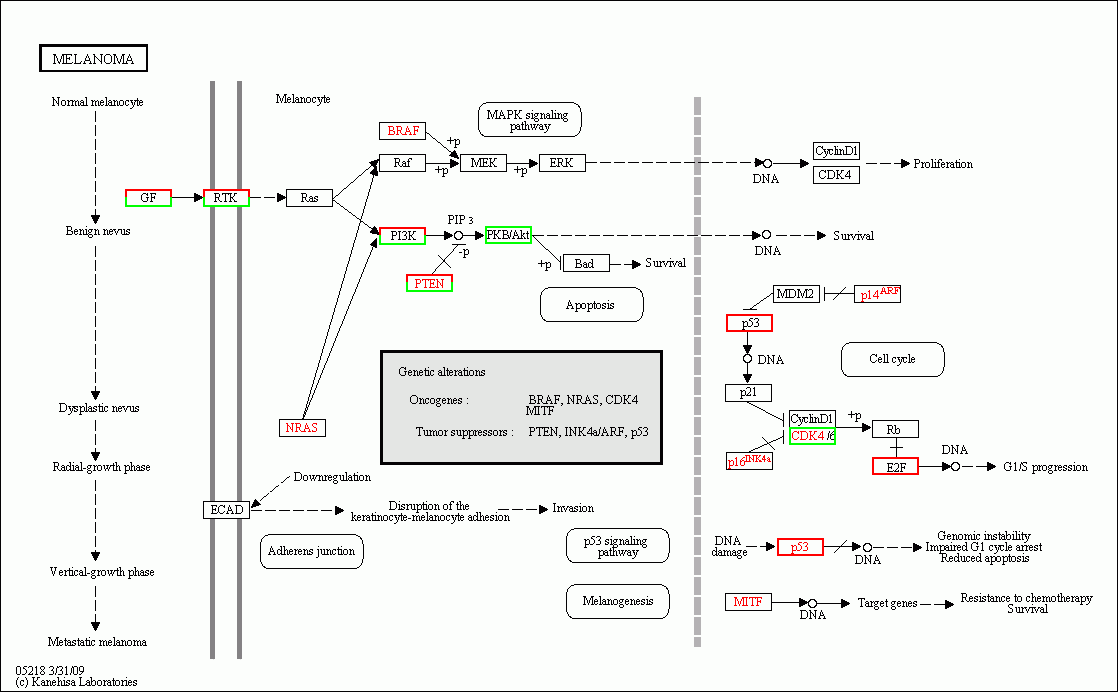


KEGG map05218: Melanoma

(L)


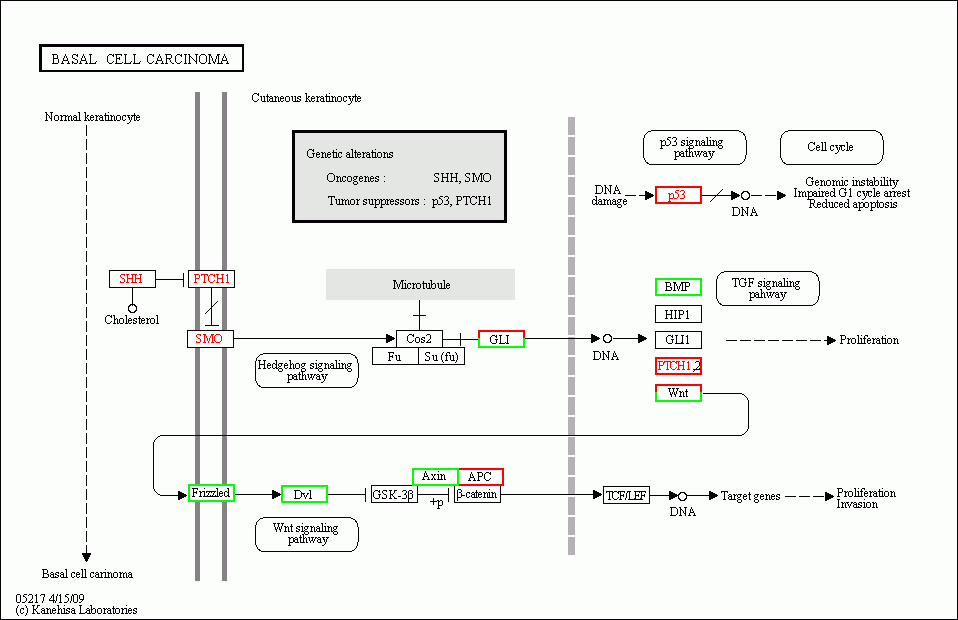


KEGG map05217: Basal cell carcinoma

(M)


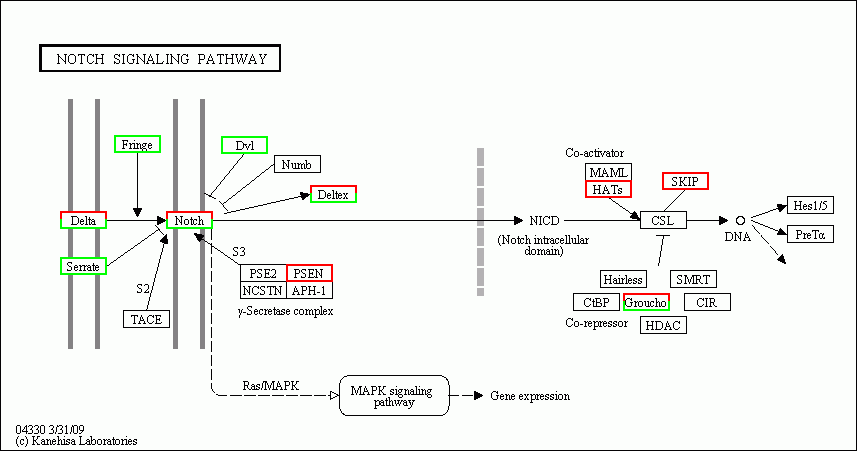


KEGG map04330: Notch signaling pathway

(N)


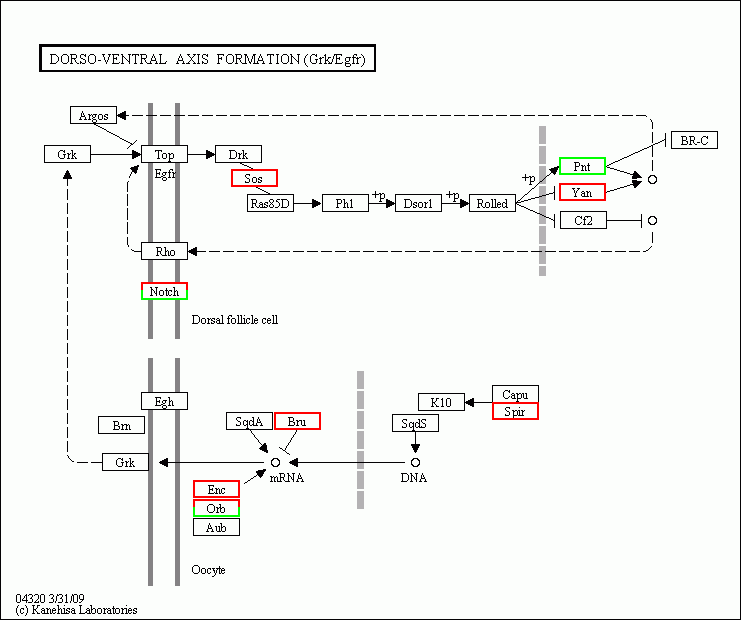


KEGG map04320: Dorso-ventral axis formation

(O)


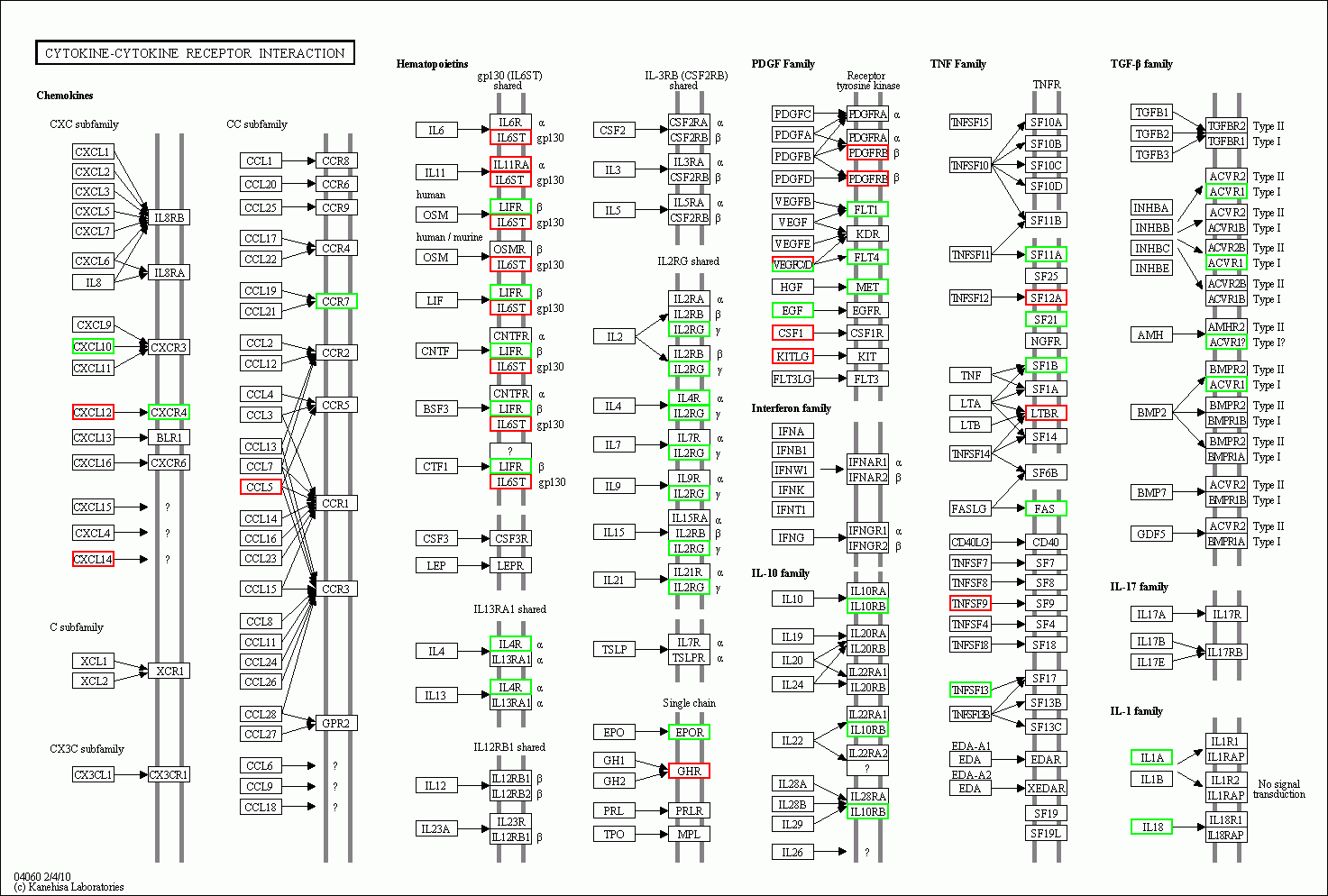


KEGG map04060: Cytokine-cytokine receptor interactions

(P)


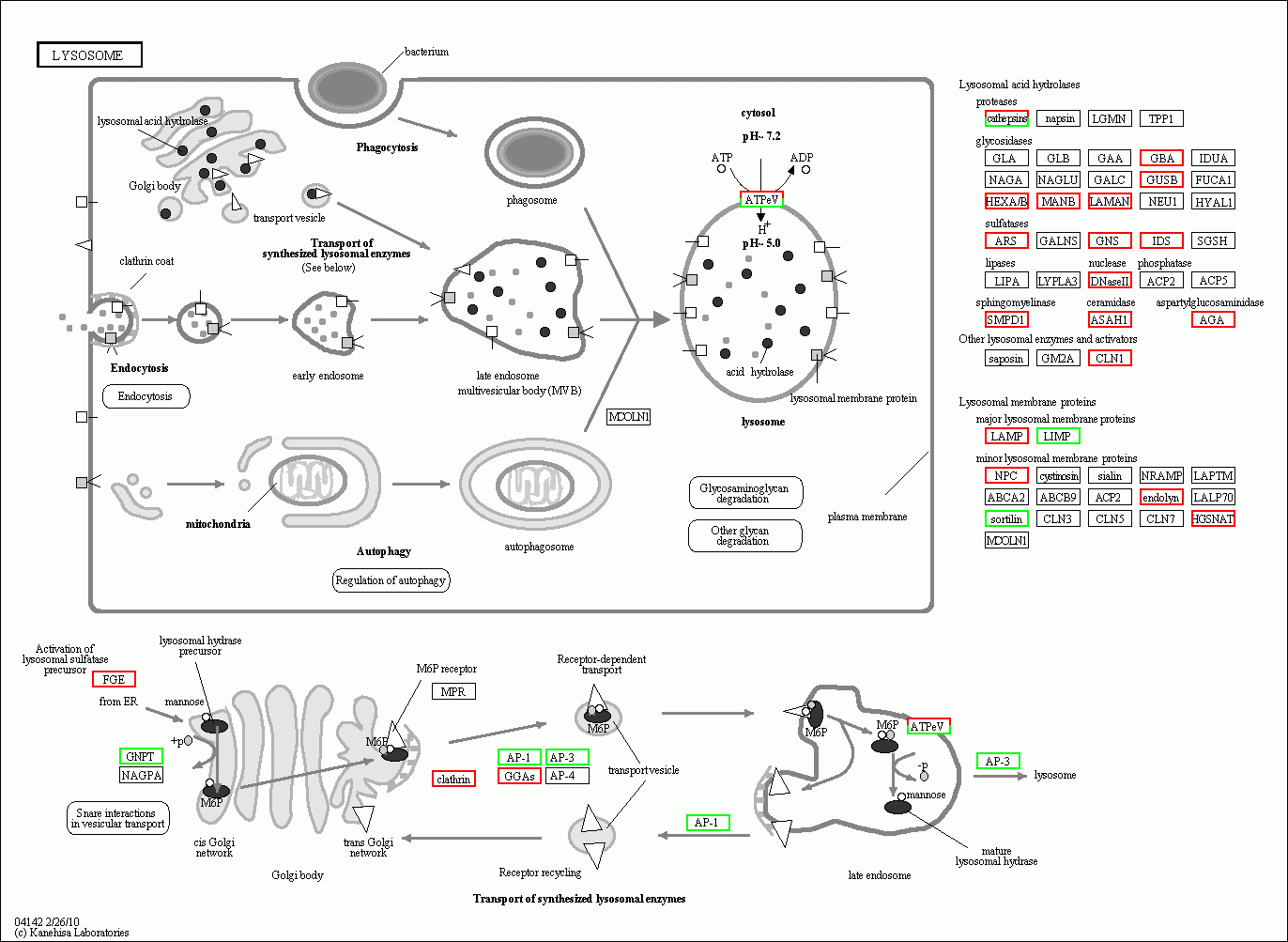


KEGG map04142: Lysosome

(Q)


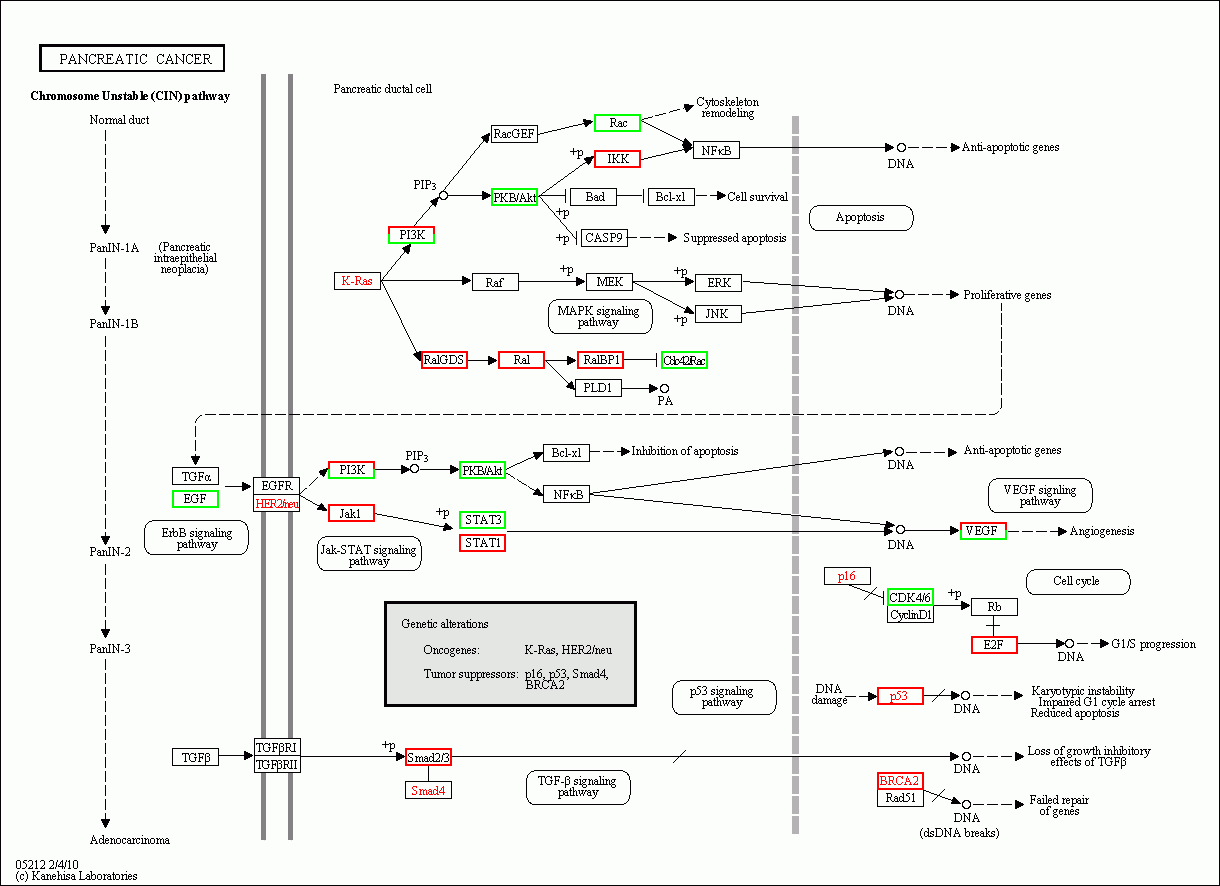


KEGG map05212: Pancreatic cancer

(R)


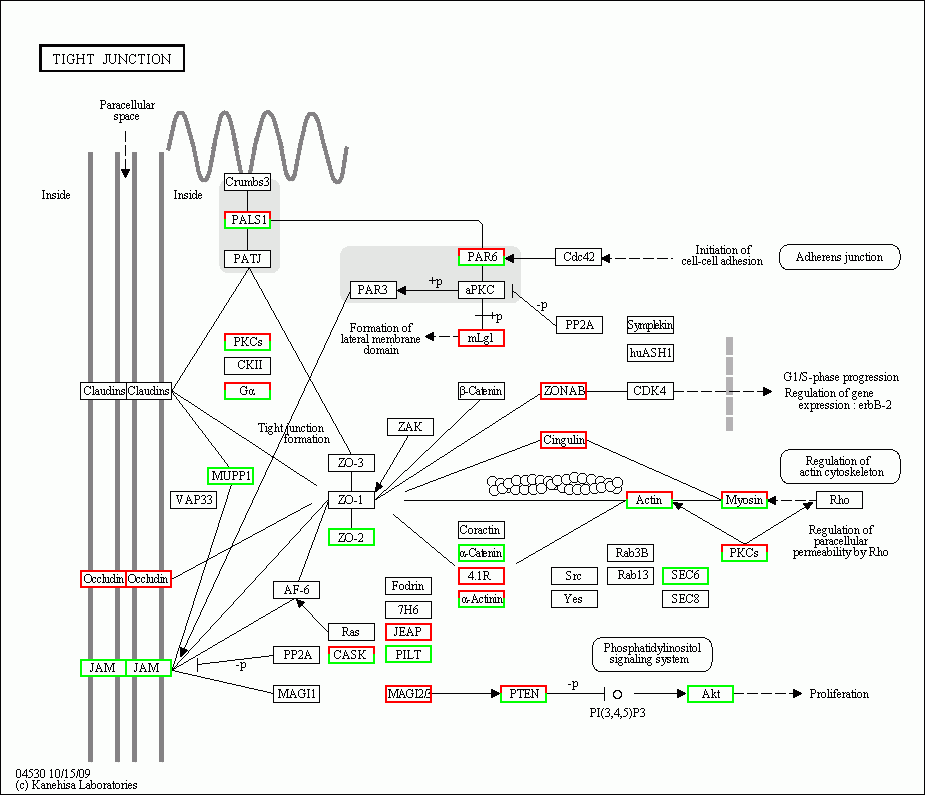


KEGG map04530: Tight junction

(S)


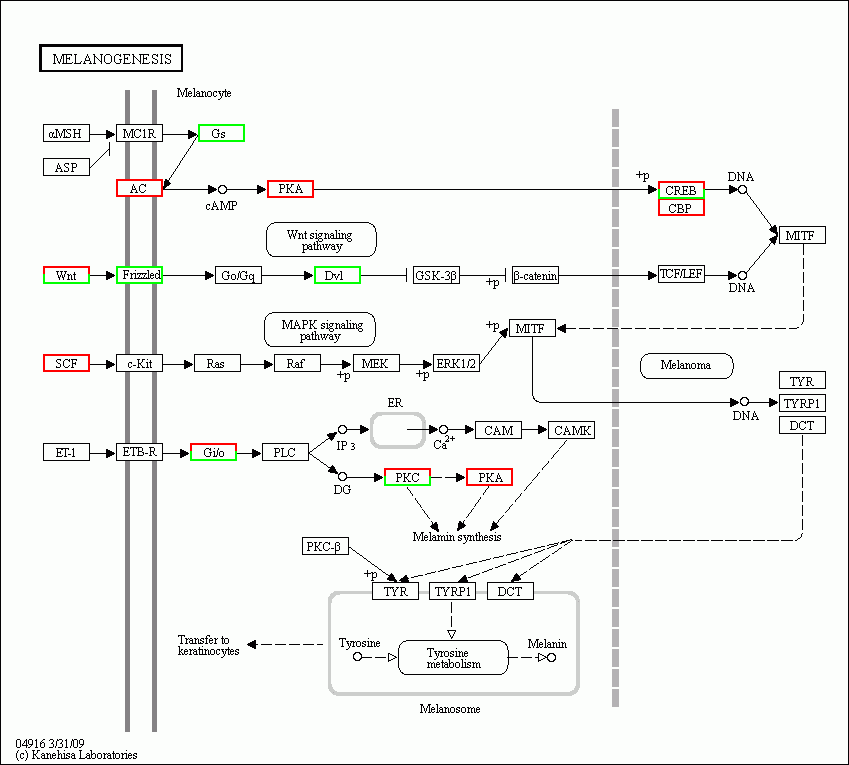


KEGG map04916: Melanogenesis

(T)


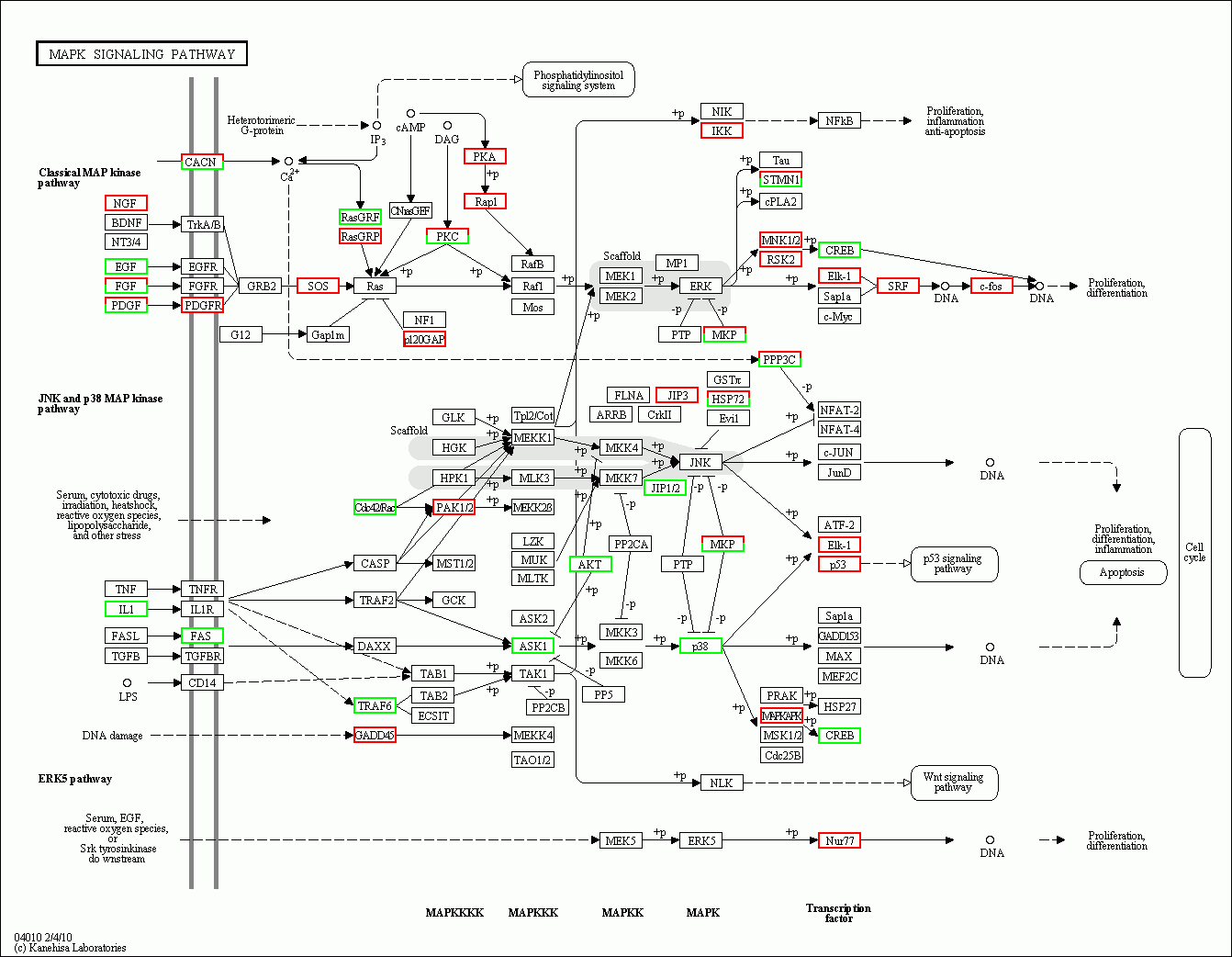


KEGG map04010: MAPK signaling pathway

(U)


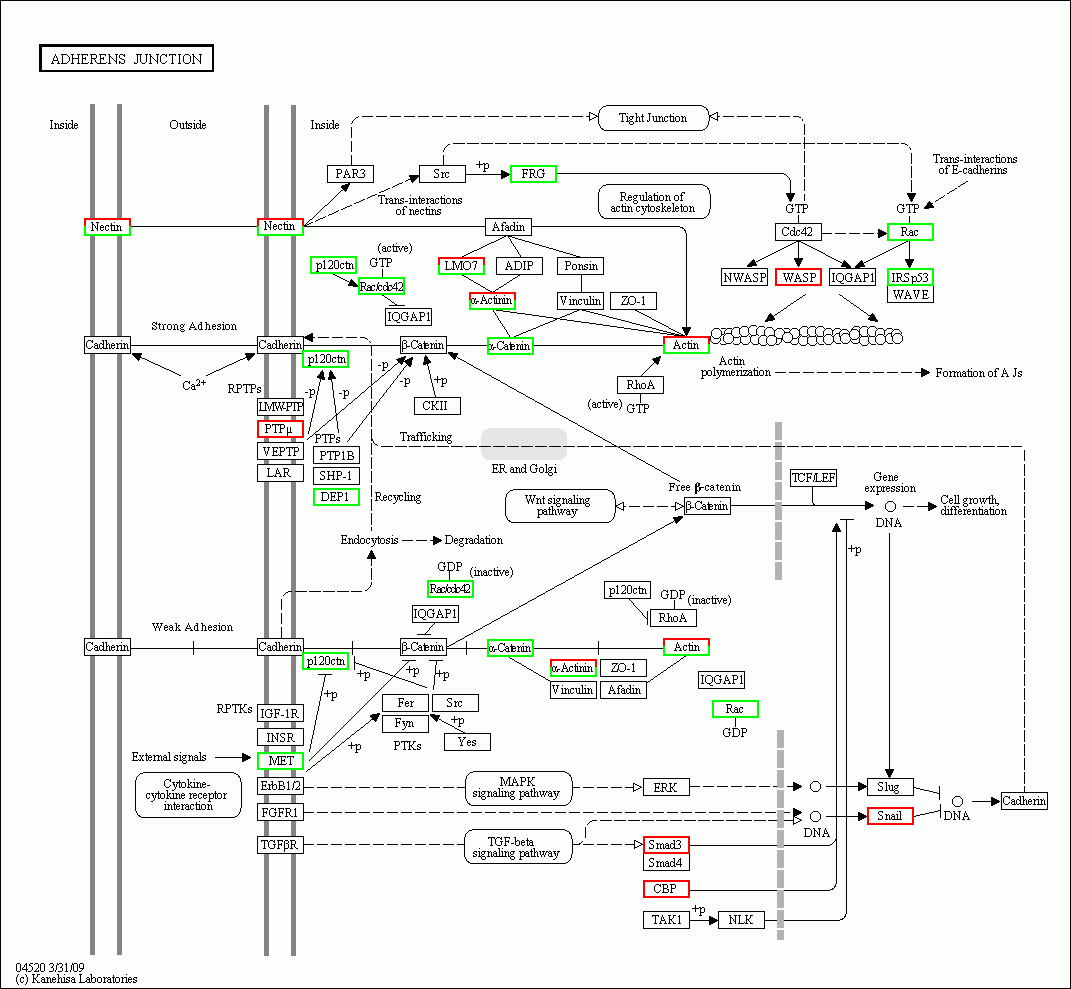


KEGG map04520: Adherens junction

**Figure S3. Significantly enriched signaling pathways of DEGs detected between GC-1spg and GC-2spd (ts).** *P* values < 0.05 and a FDR of 0.05 were selected as significant criteria for the two-sided Fisher’s exact test.
